# Molecular titanium nitrides: nucleophiles unleashed†
†Electronic supplementary information (ESI) available. CCDC 1490138–1490143. For ESI and crystallographic data in CIF or other electronic format see DOI: 10.1039/c6sc03422e



**DOI:** 10.1039/c6sc03422e

**Published:** 2016-09-22

**Authors:** Lauren N. Grant, Balazs Pinter, Takashi Kurogi, Maria E. Carroll, Gang Wu, Brian C. Manor, Patrick J. Carroll, Daniel J. Mindiola

**Affiliations:** a Department of Chemistry , University of Pennsylvania , 231 South 34th Street , Philadelphia , PA 19104 , USA . Email: mindiola@sas.upenn.edu; b Eenheid Algemene Chemie (ALGC) , Vrije Universiteit Brussel (VUB) , Pleinlaan 2 , 1050 , Brussels , Belgium; c Department of Chemistry , Queen's University , Kingston , Ontario , Canada K7L 3N6

## Abstract

In this contribution we present reactivity studies of a rare example of a titanium salt, in the form of [μ_2_-K(OEt_2_)]_2_[(PN)_2_Ti≡N]_2_ (**1**) (PN^–^ = *N*-(2-(diisopropylphosphino)-4-methylphenyl)-2,4,6-trimethylanilide) to produce a series of imide moieties including rare examples such as methylimido, borylimido, phosphonylimido, and a parent imido. For the latter, using various weak acids allowed us to narrow the p*K*_a_ range of the NH group in (PN)_2_Ti≡NH to be between 26–36. Complex **1** could be produced by a reductively promoted elimination of N_2_ from the azide precursor (PN)_2_TiN_3_, whereas reductive splitting of N_2_ could not be achieved using the complex (PN)_2_Ti=N=N=Ti(PN)_2_ (**2**) and a strong reductant. Complete N-atom transfer reactions could also be observed when **1** was treated with ClC(O)^t^Bu and OCCPh_2_ to form NC^t^Bu and KNCCPh_2_, respectively, along with the terminal oxo complex (PN)_2_Ti≡O, which was also characterized. A combination of solid state ^15^N NMR (MAS) and theoretical studies allowed us to understand the shielding effect of the counter cation in dimer **1**, the monomer [K(18-crown-6)][(PN)_2_Ti≡N], and the discrete salt [K(2,2,2-Kryptofix)][(PN)_2_Ti≡N] as well as the origin of the highly downfield ^15^N NMR resonance when shifting from dimer to monomer to a terminal nitride (discrete salt). The upfield shift of ^15^N_nitride_ resonance in the ^15^N NMR spectrum was found to be linked to the K^+^ induced electronic structural change of the titanium-nitride functionality by using a combination of MO analysis and quantum chemical analysis of the corresponding shielding tensors.

## Abstract 2

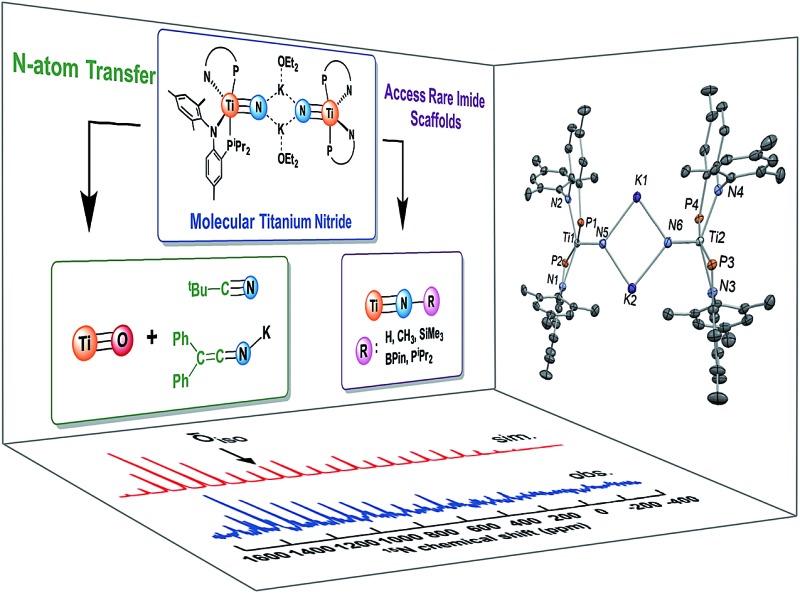
Reactivity studies of a rare example of a molecular titanium nitride are presented. A combination of theory and NMR spectroscopy provide a description of the bonding in the these nitrides, the role of the counter cation, K^+^, as well as the origin of their highly downfield ^15^N NMR spectroscopic shifts.

## Introduction

Investigation of transition metal nitrides by synthetic and physical chemists alike over the past several decades has revealed the relevance of this ubiquitous functional group to industrial and biological processes, as well as their applications in materials and surface chemistry.^1–7^ Nitride reactivity can be tuned with the appropriate transition metal ion to render this site either nucleophilic or electrophilic. High oxidation states, generally, can be stabilized with this type of motif and are known to participate in reactivity spanning various transformations^8^ such as N-atom transfer reactions and nitrile-alkyne cross-metathesis,^9,10^ and also to act as surface supports that engage in important industrial processes such as Haber–Bosch^11–14^ and hydrodenitrogenation and hydrodesulfurization.^15–19^ The nitride ligand can also provide a realistic snapshot of the active site of nitrogenases through molecular platforms modeling N_2_ reductive splitting reactions.^6,20^ Additionally, nitride-based materials are investigated for use in thin films, often derived *via* chemical vapor deposition and by direct current electron sputtering.^21–25^ Consequentially, the nitride group is extremely important in small molecule activation, in modeling and performing N_2_ to ammonia synthesis, and in producing new materials with important electronic properties or applications.

**Fig. 1:**
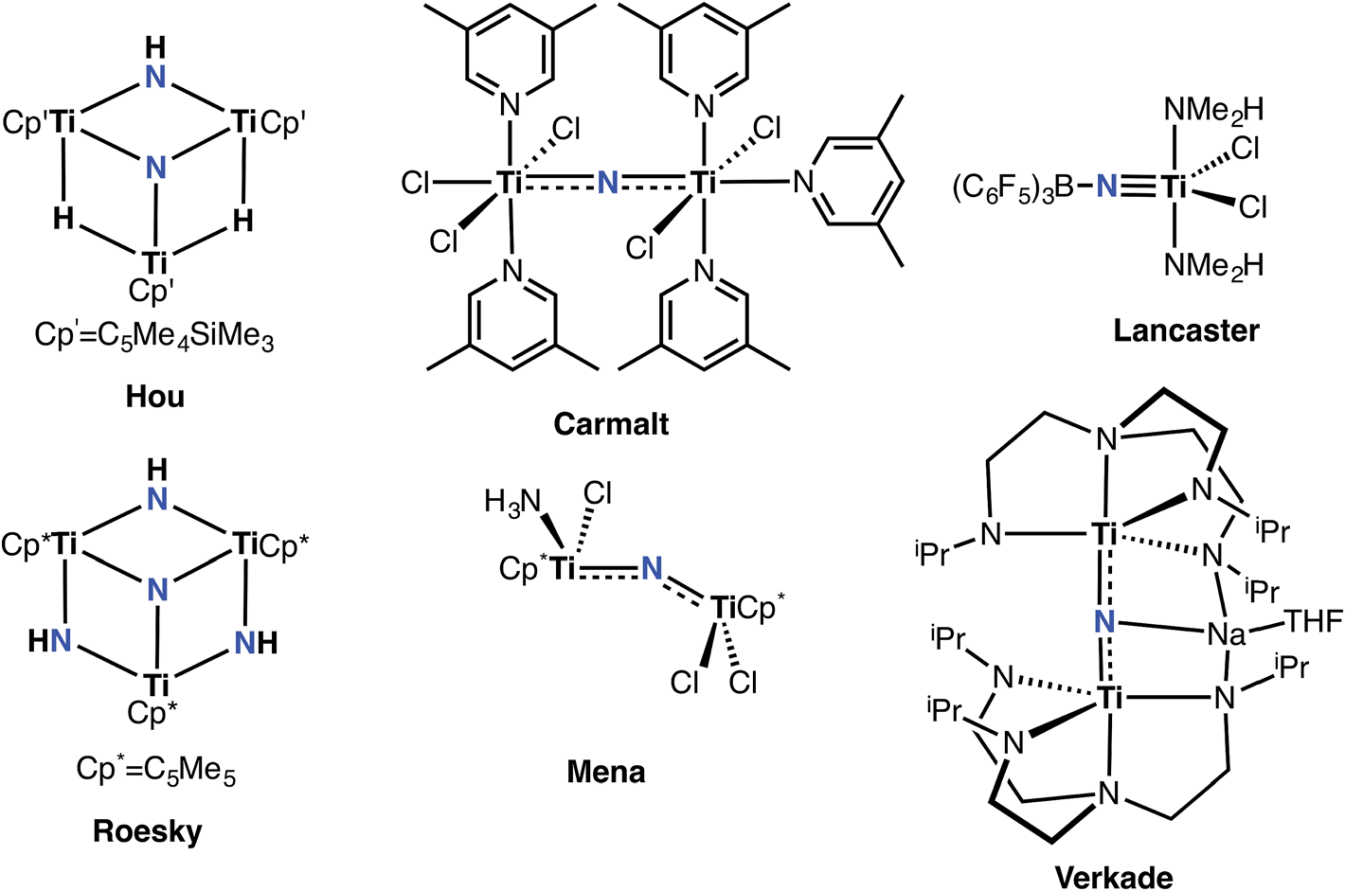
Examples of molecular titanium nitrides.

While the synthesis of both terminally bound as well as bridging metal nitrides are rather routine for groups 6–7 transition metals,^7,26–29^ a convergent synthesis of these highly nucleophilic terminal transition metal nitrides for groups 4 or 5 metals has not been documented until recently, and hence, their reactivity has been rather unexplored when compared to groups 6 and 7 derivatives.^30–41^ Of particular interest are group 4 terminally bound Ti nitrides, expected to be highly ionic given the disparity in Pauling electronegativities between Ti (1.5) and N (3.0). Only in certain cases could the nitride ligand be installed with protecting Lewis acidic groups (Fig. 1).^42–44^ However, examples of well-defined group 4 nitrides are indeed known, and can be isolated as dinuclear (Fig. 1), trinuclear (Fig. 1), tetranuclear, and even hexanuclear species.^42–51^ Thus, we focused our attention on group 4, especially on Ti, since reactivity studies of mononuclear nitrides in this group have largely evaded the reach of the synthetic chemist until recently.^52,53^ The fact that mononuclear titanium nitrides are exceedingly rare is rather surprising since group 4 nitrides have been proposed in N_2_ activation and reductive splitting reactions, and the nitride group is often generated in route to the deposition of thin metal nitride films. The elusive nature of terminal titanium nitrides, adjunct with the wealth of useful reactivity established in mid-transition metal nitrides, calls for the attention to probe the nature of this highly polarized bond and to determine the degree of nucleophilicity in this rather uncharacterized motif.

**Fig. 2:**
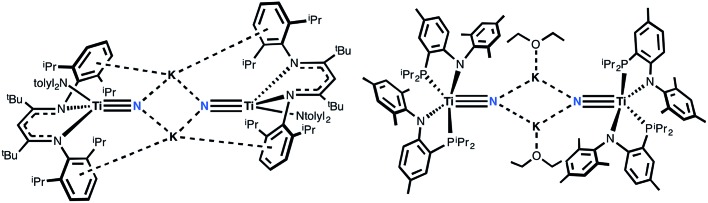
Structures of molecular titanium nitrides salts using β-diketiminate or phosphino-anilide ligands.

**Fig. 3:**
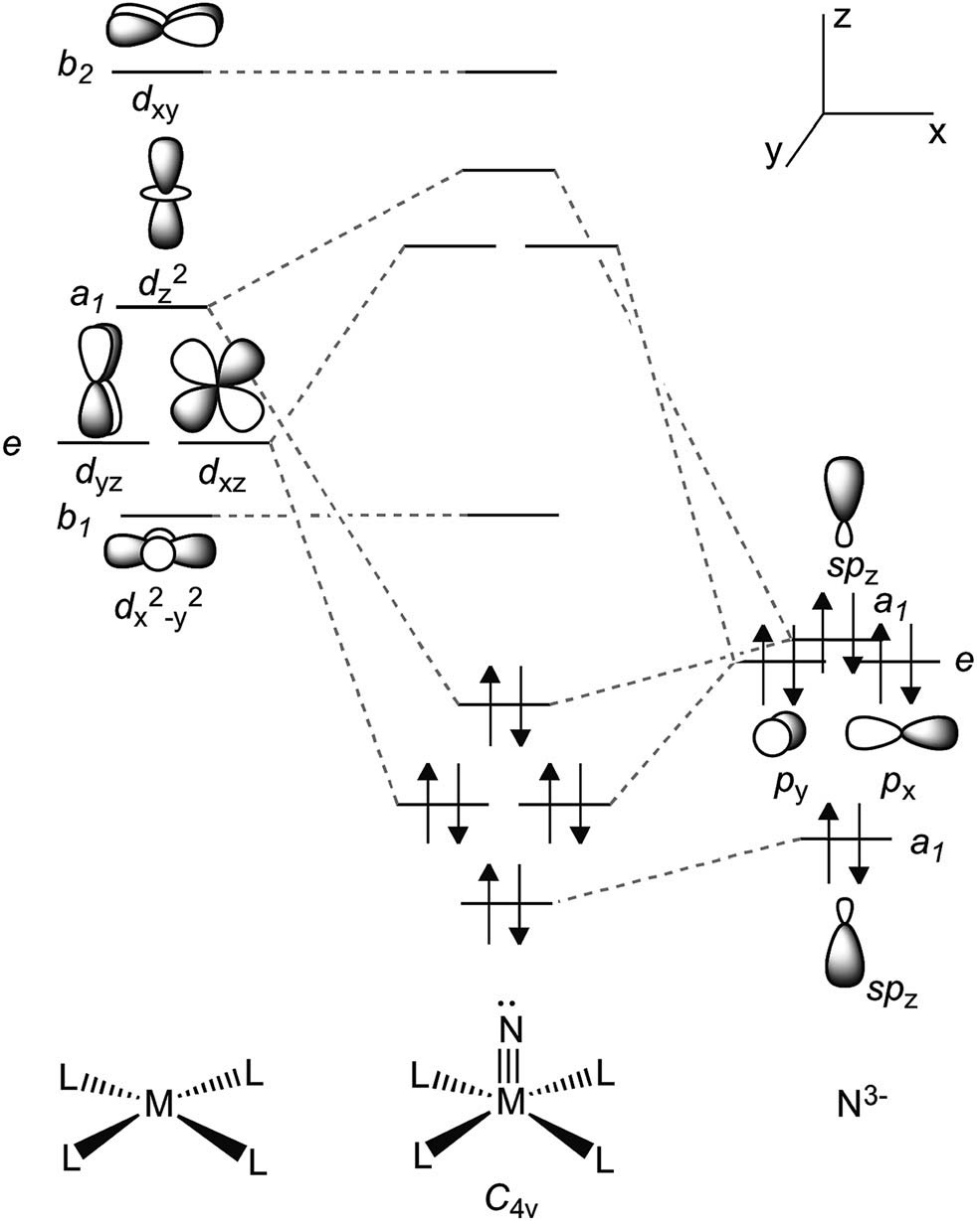
Simplified d-orbital splitting diagram for a tetragonal ML_4_ fragment (d^0^) and its compatibility with a nitride ligand N^3–^.

Recently, we reported the synthesis of a dinuclear titanium-nitride complex supported by a sterically encumbering β-diketiminate (BDI) ligand (Fig. 2).^52^ However, due to degradation in this ligand scaffold, as well as other decomposition pathways, reactivity studies of this system were quite restricted. We turned instead to new titanium nitrides supported by two PN^–^ ligands (PN^–^ = *N*-(2-(diisopropylphosphino)-4-methylphenyl)-2,4,6-trimethylanilide, Fig. 2), derived from a more direct route *via* reduction of the corresponding azide precursor.^53,54^ This ligand framework was selected as it was speculated to be more robust, lacking vulnerable protons in the ligand backbone, as well as the imine group present in β-diketiminates, which can be split under reducing conditions.^55^ Inspired by Parkin and Woo's five coordinate L_*n*_Ti=X complexes^56,57^ (L_*n*_^2–^ = *meso*-substituted porphyrin, octamethyldibenzotetraaza[14]annulene; X = chalcogen or imide group), we hypothesized that two PN^–^ ligands could enforce a pseudo tetragonal ligand field environment suitable for the construction of a terminally bound nitride or other isoelectronic atoms or groups. Fig. 3 depicts a simplified d-orbital splitting diagram for a square planar Ti^4+^ fragment supported by four σ donor nitrogen ligands, two of which could also serve as π-donors in a transoid orientation, akin to the ubiquitous porphyrin scaffold. For a d^0^ fragment, the empty and hybridized d_*z* ^2^_ as well as π-like d_*xz*_ and d_*yz*_ orbitals are available to form a triple bond with an incoming axial ligand. Further evidence that a chelating ligand such as PN^–^ could enable a more robust Ti–X multiple bond (such as a nitride) derived from the fact that terminally bound chalcogenido complexes of the Ti^4+^ ion supported by two monoanionic benzamidinate ligands could be isolated as demonstrated in the work by Arnold.^58–60^ However, unlike the highly constrained porphyrin or octamethyldibenzotetraaza[14]annulene dianion ligands, the use of two chelating ligands allows for a more flexible geometric interplay between trigonal bipyramidal and square pyramidal scaffolds when such a system is confronted by a fifth ligand.

Isolation of the dinuclear, or mononuclear titanium nitride complexes having the anionic core [(PN)_2_Ti≡N]^–^, presents an exciting opportunity to exploit such functionality, either by salt elimination followed by complete and incomplete N-atom (where the nitride is not relinquished by the metal center) transfer. Herein, we report reactivity of the dinuclear nitride complex [μ_2_-K(OEt_2_)]_2_[(PN)_2_Ti≡N]_2_ (**1**) including the access to a rare parent imide ligand, in addition to a variety of other uncommon imide species. We examine the basicity of this nitride, and we report salt elimination reactions, some of which result in complete N-atom transfer. In conjunction with solution-state spectroscopic studies of these species we also report solid state ^15^N NMR spectroscopy (*via* MAS) to further elucidate the electronic nature of the Ti–N_nitride_ bond and the effect on this bond by cation coordination. In addition, with the aid of theory we also carry out a detailed study of the Ti–N_nitride_ multiple bond and the role of the counter cation in the Ti–N_nitride_ bonding.

## Experimental section

### General procedures

Unless otherwise stated, all operations were performed in a M. Braun Lab Master double-dry box under an atmosphere of purified dinitrogen or using high vacuum standard Schlenk techniques under an argon atmosphere. NMR spectra were recorded on a Bruker AV-II 500 MHz spectrometer for ^13^C spectra, and a Bruker AVIII 400 MHz spectrometer for ^1^H and ^31^P{^1^H} spectra. ^1^H NMR spectra are reported with reference to residual proteo solvent resonances of benzene-d_6_ at 7.16 ppm. ^13^C{^1^H} NMR spectra were referenced to solvent resonances of benzene-d_6_ at 128.06 ppm. ^31^P{^1^H} NMR spectra were referenced to external H_3_PO_4_ (0 ppm). Pentane, hexanes, benzene, and toluene were purchased from Fisher Scientific and stabilizer-free diethylether (Et_2_O) and tetrahydrofuran (THF) were purchased from Sigma Aldrich. Solvents were sparged with argon for 20 minutes and dried using a two-column solvent purification system where columns designated for pentane, hexanes, benzene, and toluene were packed with Q5 and alumina respectively, and columns designated for Et_2_O and THF were packed with alumina. Deuterated benzene and deuterated toluene were purchased from Cambridge Isotope Laboratories (CIL) and were dried over 4 Å sieves, and degassed by freeze–pump–thaw cycles. All solvents were transferred into a dry box and were stored over 4 Å sieves. All sieves were heated to 200 °C under vacuum overnight prior to use. Celite used for filtrations was also heated to 200 °C under vacuum overnight prior to use. Compounds **1**, **1**-^15^N, [K(2,2,2-Kryptofix)][(PN)_2_Ti≡ ^15^N], and (PN)_2_TiCl were prepared following published procedures.^53^ The UV-Vis absorption spectra were obtained in a J-young valve 1 cm quartz cell on a Cary 5000 UV-Vis-NIR. Elemental analyses were performed at a FLASH EA 1112 Series CHN analyzer (Thermo Finnigan).

#### (PN)_2_Ti=N=N=Ti(PN)_2_ (**2**)

In a 20 mL scintillation vial, (PN)_2_TiCl (700 mg, 0.916 mmol, 1 equiv.) was dissolved in 5 mL toluene as a dark brown solution. To this solution was added a 5 mL slurry of KC_8_ (123.8 mg, 0.916 mmol, 1 equiv.) while stirring with a glass coated metal stirbar. The mixture was stirred overnight at room temperature, during which time it became a deep red color. This solution was filtered over a thick pad of celite. The product is highly insoluble, so the celite pad was washed with 80 mL of toluene in order to extract the product free from the salt. The filtrate solution was concentrated to 50 mL, and then layered with 20 mL of hexane. Storage at –35 °C overnight resulted in the formation of a magenta powder, which was isolated over a frit, followed by washing with 10 mL cold toluene (673 mg, 0.458 mmol, 49%). Single crystals suitable for X-ray diffraction could be grown from a concentrated benzene solution after one week at room temperature. ^1^H NMR (400 MHz, 25 °C, benzene-d_6_): *δ* 7.04 (br, Δ*ν*_1/2_ = 8 Hz, 2H, *meta*-Ar*H*_Mesityl_), 6.85 (dd, ^3^ *J*_H–H_ = 4.12 Hz, 1H, *meta*-Ar*H*_Tolyl_), 6.73 (s, 1H, *meta*-Ar*H*_Tolyl_), 5.85 (dd, ^3^ *J*_H–H_ = 4.10 Hz, 1H, *ortho*-Ar*H*_Tolyl_), 2.87 (s, 3H, C*H*_3Tolyl_), 2.73 (br, 1H, P–C*H*–CH_3_), 2.23 (s, 3H, *ortho*-C*H*_3Mesityl_), 1.87 (s, 3H, *para*-C*H*_3Mesityl_), 1.40 (d, ^3^ *J*_H–H_ = 4.16 Hz, 3H, P–CH–C*H*_3_), 0.54 (br, 3H, P–CH–C*H*_3_), 0.29 (sept, ^3^ *J*_H–H_ = 4.13 Hz, 1H, P–C*H*–CH_3_). ^31^P{^1^H} NMR (162 MHz, 25 °C, benzene-d_6_): *δ* 18.98 (s, 2P, *P*N). Unfortunately, the poor solubility of this complex prevented us from obtaining reliable ^13^C NMR spectra. Anal. calcd for C_94_H_130_N_6_P_4_Ti_2_ (including 1 benzene per dinuclear complex): C: 72.20, H: 8.38, N: 5.37. Found: C: 73.18, H: 9.19, N: 3.96. The loss of N_2_ in the complex resulted in a lower % of nitrogen.

#### (PN)_2_Ti≡NMe (**3**)

In a 20 mL scintillation vial, compound **1** (200 mg, 0.105 mmol, 1 equiv.) was dissolved in 5 mL toluene. To this light orange solution was added methyl iodide dropwise by microsyringe (13.3 μL, 0.210 mmol, 2 equiv.) while stirring at room temperature. The color of the solution changed during addition of methyl iodide from light orange to dark red. The solution was stirred at room temperature for 15 minutes. Volatiles were removed under vacuum, resulting in a dark red residue. The residue was triturated three times with five mL of pentane. The resulting powder was dissolved in 5 mL pentane and filtered through celite to remove the salt. Crystallization from the 5 mL filtrate solution overnight at –35 °C resulted in the isolation of red crystals (149.9 mg, 0.198 mmol, 94.6%), which were suitable for X-ray diffraction studies. ^1^H NMR (400 MHz, 25 °C, benzene-d_6_): *δ* 6.91 (br, 2H, *meta*-Ar*H*_Mesityl_), 6.78 (s, 1H, *meta*-Ar*H*_Tolyl_), 6.89 (br, Δ*ν*_1/2_ = 4 Hz, 1H, *meta*-Ar*H*_Tolyl_), 5.84 (dd, ^3^ *J*_H–H_ = 4.10 Hz, 1H, *ortho*-Ar*H*_Tolyl_), 2.63 (s, 3H, N–C*H*_3_), 2.69 (br, Δ*ν*_1/2_ = 20 Hz, 3H, P–CH–C*H*_3_), 2.23 (s, 3H C*H*_3Tolyl_), 2.17 (s, 3H, *para*-C*H*_3Mesityl_), 2.47, (br, 3H, P–CH–C*H*_3_), 0.81 (br, 3H, P–CH–C*H*_3_), 0.34 (br, 1H, P–C*H*–CH_3_). ^31^P{^1^H} NMR (162 MHz, 25 °C, benzene-d_6_): *δ* 12.48 (s, 2P, *P*N). ^13^C{^1^H} NMR (125.8 MHz, 25 °C, benzene-d_6_): *δ* 161.8 (Ar–*C*), 147.3 (Ar–*C*), 133.4 (Ar–*C*), 131.9 (Ar–*C*), 123.8 (Ar–*C*), 112.9 (Ar–*C*), 112.8 (Ar–*C*), 57.44 (N*C*H_3_), 20.9 (P*C*H(CH_3_)_2_), 20.7 (PCH(*C*H_3_)_2_), 18.4 (Ar–*C*H_3_), 16.0 (Ar–*C*H_3_). We were unable to obtain satisfactorily elemental analysis due to the thermal sensitivity of this complex.

#### (PN)_2_Ti=NTMS (**4**)

In a 20 mL scintillation vial, compound **1** (166 mg, 0.088 mmol, 1 equiv.) was dissolved in 3 mL toluene as a light orange solution. To this solution was added trimethylsilylazide dropwise *via* a microsyringe (23 μL, 0.175 mmol, 2 equiv.) while stirring. The solution rapidly turned to a deep red, along with precipitation of a solid that deposited on the walls of the vial. The volatiles were taken to dryness after 5 minutes of stirring, and the resulting red residue was triturated with 5 mL pentane resulting in a dark red powder. This powder was dissolved in 5 mL pentane, filtered through celite, and the filtrate concentrated to 3 mL. Cooling to –35 °C overnight resulted in the isolation of dark red crystals suitable for X-ray diffraction (128.1 mg, 0.157 mmol, 90.2%). ^1^H NMR (400 MHz, 25 °C, benzene-d_6_): *δ* 7.00 (s, 1H, *meta*-Ar*H*_Tolyl_), 6.89 (br, Δ*ν*_1/2_ = 8 Hz, 2H, *meta*-Ar*H*_Mesityl_), 6.80 (dd, ^3^ *J*_H–H_ = 4.12 Hz, 2H, *meta*-Ar*H*_Mesityl_), 5.81 (dd, ^3^ *J*_H–H_ = 4.56 Hz, 1H, *ortho*-Ar*H*_Tolyl_), 2.53 (br, 3H, P–CH–C*H*_3_), 2.23 (s, 3H, C*H*_3Tolyl_), 2.14 (s, 3H, *para*-C*H*_3Mesityl_), 1.02 (s, 6H, *ortho*-C*H*_3Mesityl_), 0.217 (s, 9H, NSi*Me*_3_). ^31^P{^1^H} NMR (162 MHz, 25 °C, benzene-d_6_): *δ* 13.33 (s, 2P, *P*N). ^13^C{^1^H} NMR (125.8 MHz, 25 °C, benzene-d_6_): *δ* 161.7 (Ar–*C*), 147.6 (Ar–*C*), 133.9 (Ar–*C*), 133.6 (Ar–*C*), 133.3 (Ar–*C*), 124.2 (Ar–*C*), 113.4 (Ar–*C*), 20.9 (P*C*H(CH_3_)_2_), 20.6 (PCH(*C*H_3_)_2_), 5.3 (TMS-*C*H_3_). We were unable to obtain satisfactorily elemental analysis due to the thermal sensitivity of this complex.

#### (PN)_2_Ti=NP^i^Pr_2_ (**5**)

In a 20 mL scintillation vial, compound **1** (213 mg, 0.112 mmol, 1 equiv.) was dissolved in 5 mL toluene as a light orange solution in a 20 mL vial and cooled to –35 °C. After cooling for 20 minutes, to this solution was added chlorodiisopropylphosphine dropwise *via* microsyringe (34.5 μL, 0.225 mmol, 2 equiv.) while stirring. The solution rapidly turned dark green, and salt deposited on the walls of the vial. The volatiles were taken to dryness after five minutes, and the resulting red residue was triturated with 5 mL pentane to give a dark green powder. This powder was dissolved in 5 mL pentane, filtered over celite, and concentrated to 3 mL pentane. Cooling to –35 °C overnight resulted in the isolation of large dark green hexagonal crystals suitable for X-ray diffraction (182.2 mg, 0.212 mmol, 94.1%). ^1^H NMR (400 MHz, 25 °C, benzene-d_6_): *δ* 7.05 (s, 2H, *meta*-Ar*H*_Tolyl_), 6.95 (br, Δ*ν*_1/2_ = 20 Hz, 2H, *meta*-Ar*H*_Tolyl_), 6.82 (br s, 2H, *meta*-Ar*H*_Mesityl_), 5.86 (br, Δ*ν*_1/2_ = 20 Hz, 1H, *ortho*-Ar*H*_Tolyl_), 2.95 (br, 3H, P–CH–C*H*_*3*_), 2.86 (s, 3H, C*H*_3Tolyl_), 2.21 (s, 3H, C*H*_3Tol_), 2.16 (s, 3H, *para*-C*H*_3Mesityl_), 2.12 (s, 6H, *ortho*-C*H*_3Mesityl_), 1.96 (sept, ^3^ *J*_H–H_ = 4.31 Hz, 1H, N PC*H*(CH_3_)_2_), 1.61, 1.49 (br, 3H, NPCH(C*H*_3_)_2_), 0.30 (br, Δ*ν*_1/2_ = 28 Hz, 1H, P–C*H*–CH_3_). ^31^P{^1^H} NMR (162 MHz, 25 °C, benzene-d_6_): *δ* 152.33 (br, Δ*ν*_1/2_ = 16 Hz, 1P, N*P* ^i^Pr_2_), 13.65 (br, Δ*ν*_1/2_ = 30 Hz, 2P, *P*N). ^13^C{^1^H} NMR (125.8 MHz, 25 °C, benzene-d_6_): *δ* 137.8 (Ar–*C*), 133.6 (Ar–*C*), 130.2 (Ar–*C*), 127.5 (Ar–*C*), 124.7 (Ar–*C*), 113.2 (Ar–*C*), 22.1 (NP*C*H(CH_3_)_2_), 20.9 (NPCH(*C*H_3_)_2_), 20.7 (P*C*H(CH_3_)_2_), 20.6 (PCH(*C*H_3_)_2_), 18.5 (Ar–*C*H_3_), 14.4 (Ar–*C*H_3_). We were unable to obtain satisfactorily elemental analysis due to the thermal sensitivity of this complex.

#### (PN)_2_Ti=NBCat (**6**)

In a 20 mL scintillation vial, compound **1** (297.5 mg, 0.157 mmol, 1 equiv.) was dissolved 5 mL toluene. To this light orange solution was added a 5 mL colorless toluene solution of chlorocatecholborane at room temperature (48.4 mg, 0.314 mmol, 2 equiv.). The solution immediately turned a deep green in color. After stirring at room temperature for 15 minutes, a colorless solid was observed on the walls of the vial, and all volatiles were removed under vacuum. The green residue was triturated once with 7 mL pentane, and then dissolved in 10 mL pentane. The solution was filtered through celite, and then the filtrate solution concentrated to 5 mL. Large green block crystals formed after cooling the solution overnight to –35 °C (239.5 mg, 0.278 mmol, 88.5%). Suitable crystals for single crystal X-ray diffraction studies were grown from the saturated hexane solution at –35 °C. ^1^H NMR (400 MHz, 25 °C, benzene-d_6_): *δ* 7.05 (dd, ^3^ *J*_H–H_ = 4.10 Hz, 1H, *meta*-Ar*H*_Tolyl_), 6.92 (br, Δ*ν*_1/2_ = 8 Hz, 2H, *meta*-Ar*H*_Mesityl_), 6.84 (s, 1H, *meta*-Ar*H*_Tolyl_), 6.82 (s, 2H, Ar–*H*_catechol_), 6.75 (m, ^3^ *J*_H–H_ = 8.10 Hz, 2H, Ar–*H*_catechol_), 5.87 (dd, ^3^ *J*_H–H_ = 8.21 Hz, 1H, *ortho*-Ar*H*_Tolyl_), 2.96 (s, 3H, C*H*_3Tolyl_), 2.35 (sept, ^3^ *J*_H–H_ = 4.55 Hz, 1H, P–C*H*–CH_3_), 2.26 (s, 3H, *ortho*-C*H*_3Mesityl_), 2.12 (s, 3H, *ortho*-C*H*_3Mesityl_), 2.13 (s, 3H, *para*-C*H*_3Mesityl_), 1.40 (br, 3H, P–CH–C*H*_3_), 0.80 (br, 3H, P–CH–C*H*_3_), 0.23 (sept, ^3^ *J*_H–H_ = 4.00 Hz, 1H, P–C*H*–CH_3_). ^31^P{^1^H} NMR (162 MHz, 25 °C, benzene-d_6_): *δ* 17.19 (s, 2P, *P*N). ^13^C{^1^H} NMR (125.8 MHz, 25 °C, benzene-d_6_): *δ* 161.4 (Ar–*C*), 148.8 (Cat-*C*), 145.6 (Ar–*C*), 138.5 (Ar–*C*), 134.5 (Ar–*C*), 133.6 (Ar–*C*), 131.8 (Ar–*C*), 130.8 (Ar–*C*), 129.9 (Ar–*C*), 122.0 (Cat-*C*), 113.3 (Ar–*C*), 111.9 (Cat-*C*), 111.5 (Ar–*C*), 21.5 (P*C*H(CH_3_)_2_), 21.3 (PCH(*C*H_3_)_2_), 20.9 (PCH(*C*H_3_)_2_), 20.6 (PCH(*C*H_3_)_2_), 20.0 (PCH(*C*H_3_)_2_), 17.9 (Ar–*C*H_3_), 15.9 (Ar–*C*H_3_). Unfortunately, we were unable to observe a ^11^B resonance in the ^11^B NMR spectrum. We were unable to obtain satisfactorily elemental analysis due to the thermal sensitivity of this complex.

#### (PN)_2_Ti≡NH (**7**)

Experimental note: synthesis of **7** must be conducted in the absence of light; further chemical transformations proceed in ambient light. Compound **1** (300 mg, 0.16 mmol, 1 equiv.) was dissolved in 6 mL toluene in a 20 mL vial. To this light orange solution was added a 5 mL colorless toluene solution of hexamethyldisilazane (66.3 μL, 0.32 mmol, 2 equiv.) at room temperature. Immediate color change to a magenta color was observed, and the solution was stirred for 15 minutes at ambient temperature. The solution was taken to dryness and the deep red oil was triturated with 5 mL of pentane three times. This red powder was then dissolved in 7 mL pentane, filtered over celite, and concentrated to 4 mL. Cooling overnight to –35 °C resulted in the formation of dark red microcrystals (198 mg, 0.267 mmol, 83.6%). Crystals suitable for X-ray diffraction were grown from a dilute solution of **7** in THF/pentane after 2 nights at –35 °C. ^1^H NMR (400 MHz, 25 °C, benzene-d_6_): *δ* 7.01 (dd, ^3^ *J*_H–H_ = 4.53 Hz, 1H, *meta*-Ar*H*_Tolyl_), 6.88 (br, 2H, *meta*-Ar*H*_Mesityl_), 6.80 (s, 1H, *meta*-Ar*H*_Tolyl_), 5.81 (dd, *J*_H–H_ = 4.14 Hz, 1H, *ortho*-Ar*H*_Tolyl_), 5.07 (s, 1H, Ti=N*H*), 2.41 (sept, ^3^ *J*_H–H_ = 16.0 Hz, 1H, P–C*H*–CH_3_), 2.26 (s, 3H, C*H*_3Tolyl_), 2.22 (s, 3H, *ortho*-C*H*_3Mesityl_), 2.15 (s, 3H, *para*-C*H*_3Mesityl_), 0.79 (br, 3H, P–CH–C*H*_3_), 0.29 (sept, ^3^ *J*_H–H_ = 8.11 Hz, 1H, P–C*H*–CH_3_). ^31^P{^1^H} NMR (162 MHz, 25 °C, benzene-d_6_): *δ* 15.66 (s, 2P, *P*N). ^13^C{^1^H} NMR (125.8 MHz, 25 °C, benzene-d_6_): *δ* 161.6 (Ar–*C*), 146.4 (Ar–*C*), 137.6 (Ar–*C*), 133.7 (Ar–*C*), 133.4 (Ar–*C*), 131.6 (Ar–*C*), 130.4 (Ar–*C*), 129.9 (Ar–*C*), 128.4 (Ar–*C*), 124.2 (Ar–*C*), 22.6 (P*C*H(CH_3_)_2_), 21.3 (PCH(*C*H_3_)_2_), 21.0 (PCH(*C*H_3_)_2_), 20.8 (PCH(*C*H_3_)_2_), 20.7 (PCH(*C*H_3_)_2_), 18.2 (Ar–*C*H_3_), 15.9 (Ar–*C*H_3_). We were unable to obtain satisfactorily elemental analysis due to the thermal sensitivity of this complex.

#### (PN)_2_Ti=O (**8**)

Compound **1** (203.3 mg, 0.107 mmol, 1 equiv.) was dissolved in 3 mL toluene in a 20 mL vial. To this light orange solution was added pivaloyl chloride (26.4 μL, 0.214 mmol, 2 equiv.) by microsyringe at room temperature. The solution immediately turned a bright purple color, and a white solid precipitated. The solution was stirred for 15 minutes, and then all volatiles were removed under vacuum. The residue was extracted into 10 mL ether, and filtered over celite to remove alkali. The solution was concentrated to 7 mL. Cooling overnight to –35 °C resulted in the deposition of large purple crystalline plates on the walls of the vial (152 mg, 0.205 mmol, 95.9%) some of which were suitable for X-ray diffraction. NC^t^Bu was formed as a side product of the reaction, in the same ration as **8**, measured by integration with ^1^H NMR spectroscopy. Compound **8** can alternatively be synthesized by the same experimental procedure as reported above when **1** (200 mg, 0.105 mmol, 1 equiv.) was treated in the same conditions with diphenylketene (20.5 μL, 0.210 mmol, 2 equiv.). The same work-up procedure is conducted to isolate **8** from the KNCCPh_2_ side product (118.9 mg, 0.16 mmol, 76%). ^1^H NMR (400 MHz, 25 °C, benzene-d_6_): *δ* 6.88 (br, Δ*ν*_1/2_ = 10 Hz, 2H, *para*-ArH_Mesityl_), 6.86 (s, 1H, *meta*-Ar*H*_Tolyl_), 6.81 (dd, ^3^ *J*_H–H_ = 4.12 Hz, 1H, *meta*-Ar*H*_Tolyl_), 5.82 (dd, ^3^ *J*_H–H_ = 4.14 Hz, 1H, *meta*-Ar*H*_Tolyl_), 2.79 (br, 3H, P–CH–C*H*_3_), 2.26 (s, 3H, C*H*_3Tolyl_), 2.19 (s, 3H, *para*-C*H*_3Mesityl_), 1.54 (d, 3H, P–CH–C*H*_3_, *J* value could not be determined due to overlaps), 1.26 (d, 3H, P–CH–C*H*_3_, *J* value could not be determined due to overlaps), 0.89 (s, 3H, *ortho*-C*H*_3Mesityl_), 0.78 (s, 3H, *ortho*-C*H*_3Mesityl_), 0.29 (sept, ^3^ *J*_H–H_ = 8.23 Hz, 1H, P–C*H*–CH_3_). ^31^P{^1^H} NMR (162 MHz, 25 °C, benzene-d_6_): *δ* 13.33 (s, 2P, *P*N). ^13^C{^1^H} NMR (125.8 MHz, 25 °C, benzene-d_6_): *δ* 161.9 (Ar–*C*), 145.3 (Ar–*C*), 138.3 (Ar–*C*), 134.2 (Ar–*C*), 133.7 (Ar–*C*), 131.7 (Ar–*C*), 130.5 (Ar–*C*), 124.7 (Ar–*C*), 113.1 (Ar–*C*), 20.9 (P*C*H(CH_3_)_2_), 19.6 (PCH(*C*H_3_)_2_), 15.6 (Ar–*C*H_3_), 15.9 (Ar–*C*H_3_). We were unable to obtain satisfactorily elemental analysis for this complex.

## Results and discussion

**Scheme 1:**
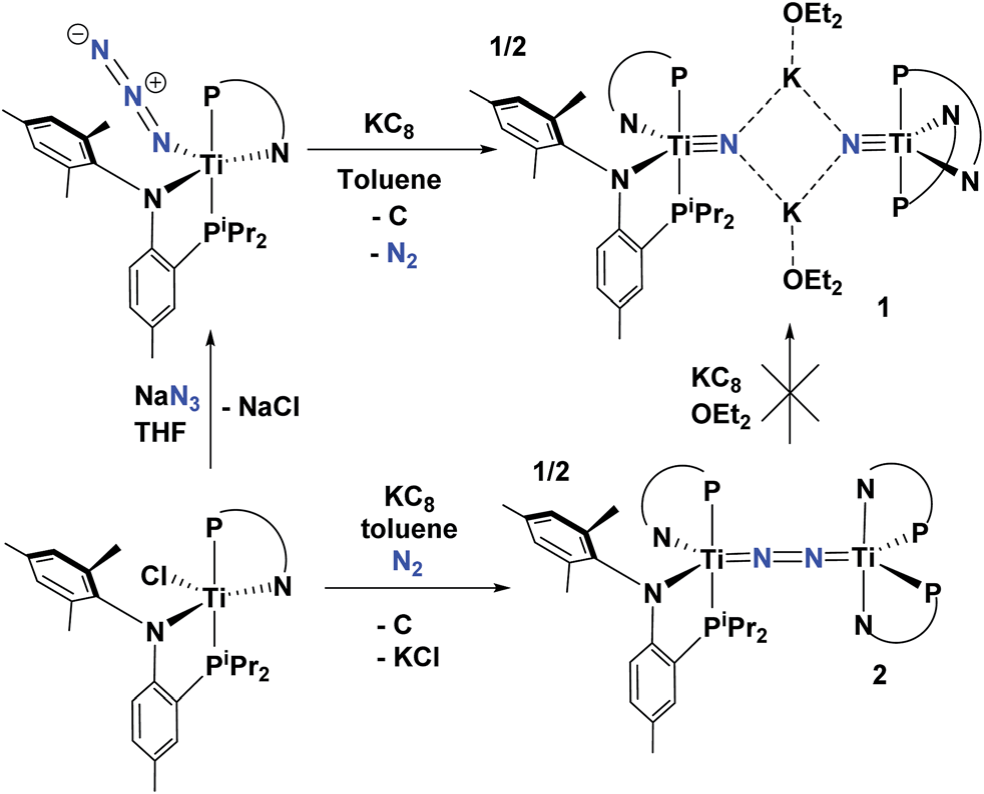
Synthesis of complex **1** from reductive splitting of an azide with KC_8_. Also shown is the attempted synthesis of **1** from reductive splitting of N_2_ in **2** with excess KC_8_.

**Fig. 4:**
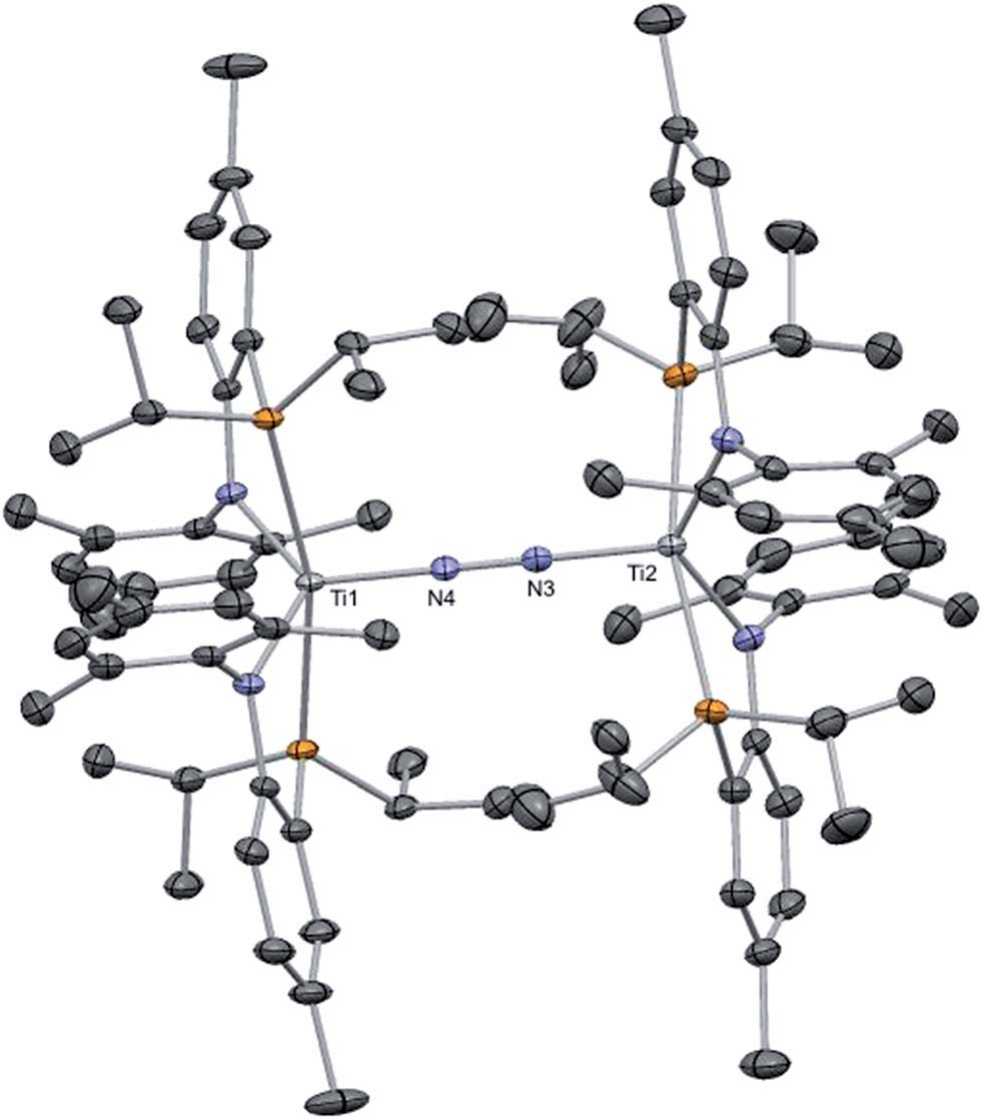
Solid state structure of complex **2** showing thermal ellipsoids at the 50% probability level. Selected metrical parameters (distances Å in and angles in °) are shown in Table 1. H atoms and a residual benzene found in the asymmetric unit have been omitted for clarity.

Recently, we reported that the azide complex (PN)_2_Ti(N_3_), readily prepared from (PN)_2_TiCl and NaN_3_, could undergo reductive extrusion of N_2_ with KC_8_ in Et_2_O to form the nitride salt [μ_2_-K(OEt_2_)]_2_[(PN)_2_Ti≡N]_2_ (**1**) along with graphite (Scheme 1).^53^ This route circumvents a radical mechanism commonly observed in the formation of the parent imido (^tBu^BDI)Ti≡NH(Ntolyl_2_),^52^ which results in much lower yield due to the sacrificial H-atom source deriving from the ligand scaffold. In addition, the introduction of the nitride group directly from reduction of the azide skips an additional deprotonation step involving the parent imido. To our surprise however, we found that the dinitrogen complex (PN)_2_Ti=N=N=Ti(PN)_2_ (**2**), a species prepared in 49% yield from KC_8_ reduction of (PN)_2_TiCl under N_2_, cannot be fragmented with excess reductant (such as KC_8_) to form two equivalents of complex **1**. This route would undoubtedly provide a more atom economical route to **1** using a vast resource such as atmospheric N_2_. Complex **2** is a diamagnetic species, displaying one single PN chemical environment by both ^1^H and ^31^P NMR spectra, and as shown in Fig. 4, its solid state structure reveals a topologically linear TiN_2_Ti moiety where the N–N bond has been partially reduced by 2e^–^ (1.252(8) Å *versus* 1.0976 Å in free N_2_). Although a formal N_2_^4–^ ligand would intuitively account for its diamagnetic nature, the observed N–N and Ti–N distances and computed bond orders argue for **2** possessing two Ti(iii) centers that strongly antiferromagnetically couple, *i.e.* its core can be best characterized formally as Ti=N=N=Ti. In stark contrast to **2**, Fryzuk and co-workers have observed N_2_ splitting reactions with low-valent titanium reagents, *via* reduction of [NPN]TiCl_2_ [NPN^2–^ = PhP(CH_2_SiMe_2_NPh)_2_], to produce transient titanium nitrides which then undergo insertion into the Ti–P linkages of the ligand.^61^ For us, we attribute the lack of reactivity in **2** to being reduced is likely to be kinetic as well as thermodynamic in nature. Akin to vanadium(ii) dinitrogen complexes prepared in our group, we propose the rigid PN ligands in a putative species such as **2** ^2–^ (**2** ^2–^ represents **2** being reduced by two electrons) to disfavor the mixing of triplet and singlet subspaces that is critical for the dinitrogen cleavage process.^62^ In addition, the reactive nature of **1** suggests the formation of a nitride from N_2_ to be thermodynamically less stable than **2** ^2–^, a common trait also observed in vanadium nitrides *versus* dinitrogen complexes. Compound **2** is remarkably stable, failing to react with electrophiles as well as reductants, and further attempts to functionalize the N_2_ have been unsuccessful. Arnold and co-workers have observed formation of a similar complex to **2** using benzamidinate ligands.^59^

### Reactivity studies of the titanium nitride with electrophiles

**Scheme 2:**
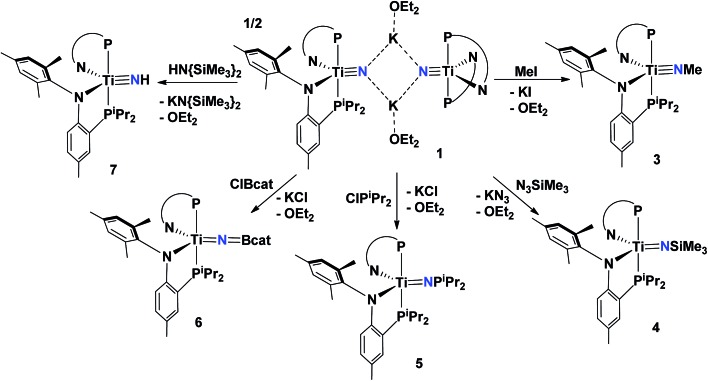
Synthesis of compounds **3–7** from **1** and various electrophiles. All reactions were performed in toluene at 25 °C due to the insolubility of **1** in aliphatic solvents.

Although bridging derivatives of group **4** methyl imides have been reported,^63,64^ terminally bound examples are unknown. Accordingly, we treated compound **1** with MeI in toluene at 25 °C causing an immediate color change from orange to dark red. Workup of the reaction mixture and recrystallization of the solid from pentane at –35 °C allowed for the isolation of the methyl-imide complex (PN)_2_Ti≡NMe (**3**) in ∼95% yield (Scheme 2). The ^31^P NMR spectrum of **3** features a singlet at 12.45 ppm, shifted slightly upfield from **1** (7.23 ppm), while the methyl imide resonance is observed at 2.63 ppm in the ^1^H NMR spectrum and correlated to a resonance at 57.4 ppm in the ^13^C NMR spectrum. A solid state structural analysis confirmed the monomeric nature of **3** (Fig. 6), and revealed a short Ti–N distance of 1.709(3) Å with a linear Ti–N–Me angle of 178.5(4)° (Table 1). While the geometry of **1** (*τ*_5_ = 0.529) is between idealized trigonal bipyramidal (*τ*_5_ = 1) and square pyramidal (*τ*_5_ = 0), conversion to the imide species **3** (*τ*_5_ = 0.721) alters the geometry more towards a trigonal bipyramidal environment.

**Table 1:** Selected metrical parameters for complexes **1–7** showing bond distances in Å and angles in degrees. X represents the substituent on the nitride or formal nitride ligand. For **1**, X = K; **2**, X = N; **3**, X = C; **4**, X = Si; **5**, X = P; **6**, X = B; **7**, X = H. *τ*_5_ represents (*β* – *α*)/60 where *β* > *α* are the two greatest valence angles of the coordination center^85^

Complex	**1**	**2**	**3**	**4**	**5**	**6**	**7**
Ti–N	1.674(2)	1.832(3)	1.709(3)	1.730(2)	1.753(3)	1.7312(2)	1.747(2)
Ti–N_PN_	2.173(2)	2.107(3)	2.093(3)	2.095(2)	2.0695(2)	2.0521(1)	2.0605(1)
Ti–N_PN_	2.170(2)	2.091(2)	2.096(3)	2.0736(2)	2.0695(2)	2.0685(1)	2.0987(2)
Ti–P	2.6779(8)	2.6763(8)	2.6777(1)	2.6917(7)	2.7041(6)	2.6718(5)	2.6810(5)
Ti–P	2.6853(8)	2.7306(8)	2.6824(1)	2.7094(7)	2.7041(6)	2.6532(6)	2.6656(1)
N–X	2.729(2)	1.250(4)	1.431(5)	1.737(2)	1.732(3)	1.395(2)	0.860(0)

P–Ti–P	167.42(3)	166.03(4)	173.14(3)	167.14(3)	171.16(3)	175.289(2)	177.16(3)
Ti–N–X	135.40(1)	1.250(4)	178.5(4)	168.06(1)	162.21(6)	175.00(1)	180.0(0)
N_PN_–Ti–N_PN_	135.67(8)	128.79(1)	129.87(1)	134.35(8)	130.02(1)	131.33(6)	130.99(6)
P–Ti–N_PN_	74.88(6)	76.30(7)	75.24(8)	73.77(6)	75.88(5)	75.27(4)	74.94(4)
P–Ti–N_PN_	75.11(6)	75.18(7)	75.13(8)	75.46(5)	75.88(5)	76.18(4)	75.70(5)
P–Ti–N	95.83(8)	96.99(2)	93.46(1)	99.59(7)	94.419(2)	92.67(5)	92.52(6)
P–Ti–N	96.75(8)	98.31(2)	93.37(1)	96.25(7)	94.419(2)	91.96(5)	90.22(7)
*τ*_5_	0.529	0.621	0.721	0.557	0.686	0.733	0.769

Treatment of **1** with certain azide reagents can also result in salt elimination. For example, combining **1** with N_3_SiMe_3_ yields KN_3_ along with the trimethylsilylimide (PN)_2_Ti=N{SiMe_3_} (**4**) in ∼90% yield as a deep red colored material (Scheme 2). Complex **4** shows similar spectroscopic features to **3** with the trimethyl resonance being observed at 0.22 ppm in the ^1^H NMR spectrum. An IR spectrum of the reaction mixture confirms the formation of KN_3_ (*ν*_N_3__ = 2030 cm^–1^). The synthesis of complex **4** was also confirmed by monitoring reactivity of **1** with Me_3_SiCl by ^1^H NMR spectroscopy. To probe the reaction mechanism with Me_3_SiN_3_, a ^15^N enriched sample of **1**, [μ_2_-K(OEt_2_)]_2_[(PN)_2_Ti≡ ^15^N]_2_ (**1**)-^15^N was prepared and treated with Me_3_SiCl to independently prepare (PN)_2_Ti= ^15^N{SiMe_3_} (**4**)-^15^N. As expected, a resonance at 534 ppm in ^15^N NMR was attributable to the imide isotopomer species **4**-^15^N. A further experiment of **1**-^15^N with Me_3_SiN_3_ reproduced the signal for **4**-^15^N in the ^15^N NMR spectrum, showing that this reaction is proceeding by salt elimination rather than by cycloaddition and a silyltropic shift. This mechanism follows the predicted behavior in reactivity with regard to the nucleophilic nitride attacking at the electrophilic silyl group of the azide. Consistent with our hypothesis, there was no reactivity between **1** and adamantyl azide or trisylazide. The solid-state structure of **4** shows an overall similar geometry to **3** but where the Ti–N distance has now been elongated to 1.730(2) Å, consistent with the presence of an electrophilic SiMe_3_ group. This is similar to reported Ti–N distances in Ti=NSiMe_3_ functionalities,^65–69^ although some shorter derivatives have been reported to be as low as ∼1.6 Å.^70^ The Ti–N–Si is slightly bent at nitrogen at 168.06(1)° (Fig. 6, Table 1). In stark contrast to **3**, the geometry of **4** remains quite similar to **1**, confined between trigonal bipyramidal and square pyramidal (*τ*_5_ = 0.557).

**Fig. 5:**
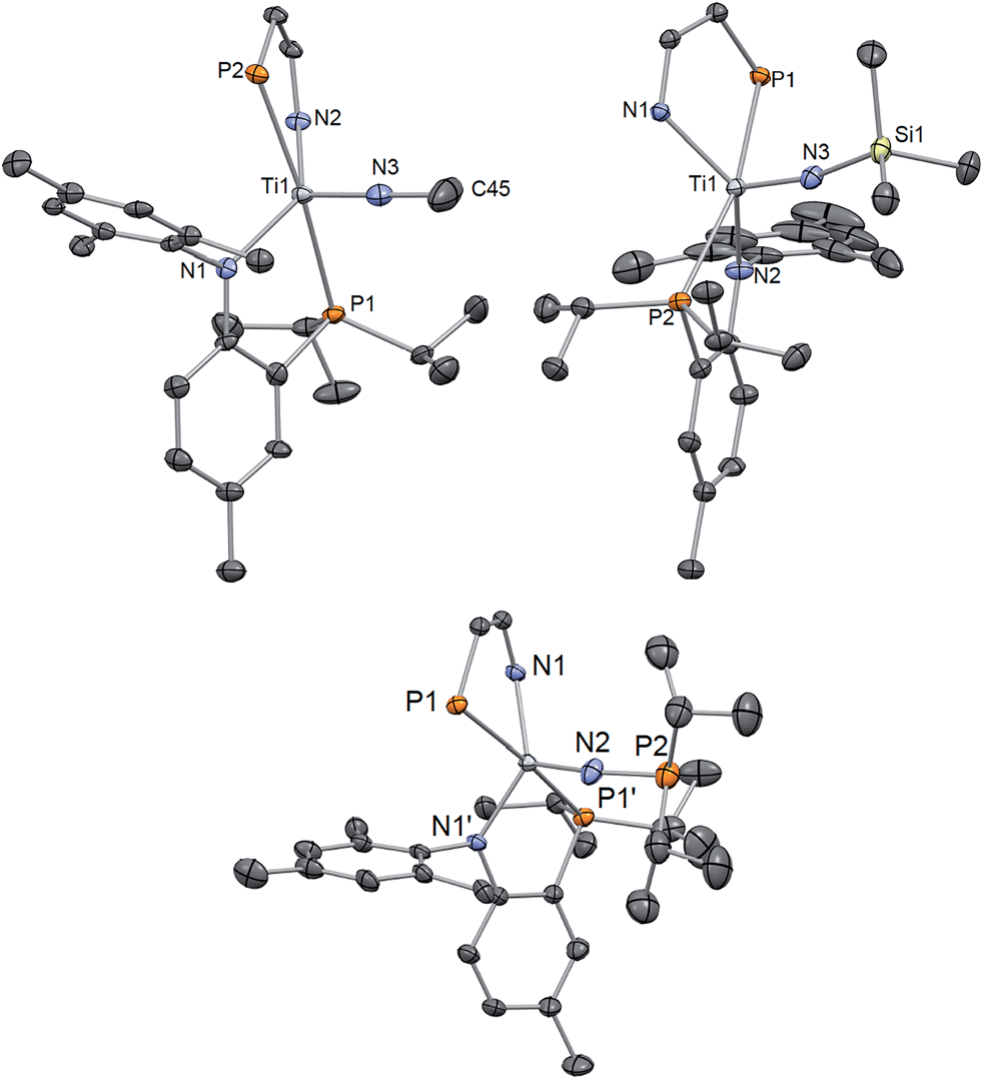
Solid state X-ray structure of complexes **3–5** depicting thermal ellipsoids at the 50% probability level. H atoms and the substituents of one depicted PN ligand have been omitted for clarity. Residual solvent molecules have been also omitted.

In pursuit of other rare functional imide groups we treated complex **1** with ClP^i^Pr_2_. Upon addition of the electrophile, the reaction mixture changed color from orange to dark green. Workup of the reaction allowed for isolation of a green colored solid in 94.1% yield and ^1^H, ^31^P and ^13^C NMR spectral data were consistent with formation of the phosphonylimide (PN)_2_Ti=N{P^i^Pr_2_} (**5**) (Scheme 2). Phosphonylimides of early transition metals are rare functionalities, with only a couple of reported examples.^71,72^ The ^31^P NMR spectrum features a broadened resonance (Δ*ν*_1/2_ = 16 Hz) attributable to the phosphonylimide at 152 ppm. Interestingly, the resonance for the P of the PN ligand appears as two broadened features centered at 13.73 ppm Δ*ν*_1/2_ = 30 Hz. Although quite broad, it is clear that these two signals account for two separate PN ligand resonances, indicating a solution-state behavior that renders these two resonances inequivalent. Additionally, when cooled, the broadened resonances slightly sharpen although P–P coupling with the phosphonylimide was unresolved, as shown in Fig. S19 (see ESI†), with a sample measured at 219 K in toluene-d_8_. Other low temperature NMR spectra are also reported in the ESI.† ^73^ We propose that, unlike other imides reported in these studies, the phosphoylimide results in inequivalency in the PN ligands due to steric clash of ^i^Pr groups of the phosphonylimide with the ^i^Pr groups of the PN ligand. The molecular structure of **5** features a Ti–N–P angle of 162.21(6)° and elongation of the Ti–N bond to 1.753(3) Å (Fig. 5, Table 1). This latter metrical parameter is similar to hydrazido complexes reported by Mountford and Odom, with Ti=N distances varying from 1.703–1.76 Å. Shorter Ti–N distances have been reported in some instances, attributable to NR_3_^+^ groups on the imido moiety.^74–78^ Complex **5** crystallizes in space group monoclinic *I*2/*a*, and the molecule lies along a two-fold axis. Consequently, this two-fold axis in turn causes disorder in the ^i^Pr groups of the phosphonyl group. Hence, we restrain ourselves from discussing metrical parameters in detail. However, the gross solid-state structure of **5** reveals a bent TiN^i^Pr_2_ moiety thus rendering both phosphorus groups on the PN ligands inequivalent (Fig. 5).

**Fig. 6:**
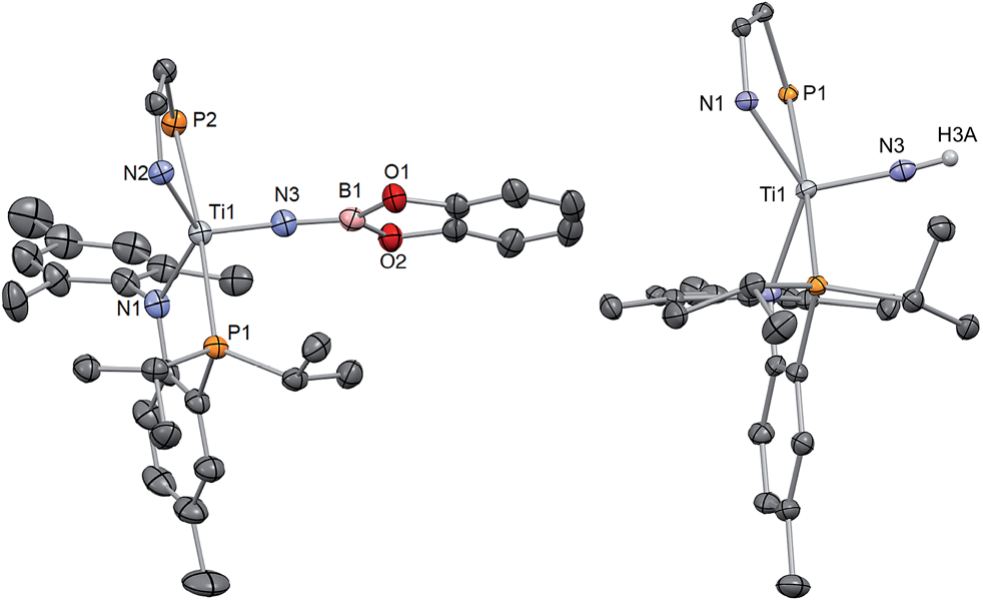
Solid state X-ray structure of complexes **6** and **7** depicting thermal ellipsoids at the 50% probability level. H atoms and the substituents of one depicted PN ligand have been omitted for clarity. For **7**, SQUEEZE was used to remove a disordered THF molecule.

Early-transition borylimidos are rare functional groups in inorganic chemistry. Only two examples of titanium have been reported, both being derived from non-conventional routes involving B–B, B–H, and B–C bond activation reactions.^52,79^ Our ability to prepare nitride **1** allows us to explore simple salt-metathesis reactions to construct these archetypal functionalities. Hence, treating **1** with ClBcat (cat = catechol) resulted in clean conversion to the borylimido (PN)_2_Ti=N{Bcat} (**6**) in ∼89% yield after subsequent workup of the reaction mixture (Scheme 2). Unlike the phosphonylimide, the borylimido does not display any unusual NMR spectroscopic features, with a ^31^P NMR spectrum featuring one singlet resonance at 17.19 ppm. A solid-state structural study of **6** shows a topologically linear Ti=N=B (175.00(1)°) due to the Lewis acid nature of the boryl group. Consequently, the former titanium nitride distance Ti–N has elongated to 1.7312(2) Å, akin to **4** (Fig. 6, Table 1). In accord with these other rare examples, the linear character of the Ti=N=B bond angle demonstrates delocalized π electrons in these bonds.

Terminally bound imides with a hydrogen substituent (referred to as a parent imide) are exceptionally rare in early transition metals, with the only documented examples for group 4 being (BDI)Ti≡NH(Ntolyl_2_)^52,80,81^ (BDI^–^ = [ArNCMe]_2_CH or [ArNC^t^Bu]_2_CH; Ar = 2,6-^i^Pr_2_C_6_H_3_), and *trans*-TiCl_2_(NH)(OPPh_3_)_2_.^81^ Not surprisingly, p*K*_a_ information on parent imides is unknown, but one would expect the imide moiety to be a weak acid given the highly polarized nature of the nitride group. Conversely, the basicity of terminal nitride anions is also unknown. While these species have rarely been reported, it has been hypothesized that some parent imide complexes exist as transient, non-isolable intermediates.^14^ Since formation of **1** does not traverse through a parent imide, we treated this species with a weak acid not only to allow us entry to this rare moiety but also to provide some information about the basicity of the nitride ligand in **1**. Hence, exploring various sources of a proton established HN{SiMe_3_}_2_ (p*K*_a_ = 25.8, THF)^82^ to cleanly yield the parent imide (PN)_2_Ti≡NH (**7**) in ∼84% isolated yield (Scheme 2). The imide resonance was observed in the ^1^H NMR spectrum as a broad feature at 5.07 ppm (Δ*ν*_1/2_ = 9.1 Hz) while the ^31^P NMR spectrum displayed a singlet at 15.66 ppm, a resonance shifted downfield from **1**. Characterization by single X-ray crystallography (Fig. 6) revealed that complex **7** favors a more trigonal bipyramidal geometry, (*τ*_5_ = 0.769), which contrasts the geometry observed for the few known group 4 transition metal parent imides having coordination numbers of four and five.^81^ One notable feature is the nearly linear orientation of the phosphine groups (P–Ti–P, 177.16(3)°) when associated to other (PN)_2_Ti scaffolds (Table 1). It is also noted that single X-ray diffraction revealed that the molecule also co-crystallizes in a 4 : 1 ratio with a secondary product in which the N atom of the imide has inserted into the phosphorous arm of the PN ligand, and the H atom of the parent imide has formed a bond to titanium, namely the compound “(PN)(NPN)Ti(H)” (see ESI† for an explanation of these co-crystals). The metal-hydride bond that is observed as a result of this transformation is under further investigation with regard to the implications this rearrangement has on reactivity of the parent imide. While the ylide Ph_3_PCH_2_ (p*K*_b_ = –22, DMSO)^83^ failed to deprotonate the imide, attempts to use stronger bases resulted in decomposition products presumably due to the reactivity of the titanium nitride product. It was found independently that complex **7** gradually decomposes over time thus precluding reactivity with stronger bases. However, attempts to protonate the nitride with diisopropylamine, HN^i^Pr_2_ (p*K*_a_ = 36, THF),^84^ resulted in no reaction. This observation allowed us to narrow the p*K*_a_ range of the parent imide to be within 26–36 based on these set of control experiments. Given these p*K*_a_ ranges for the parent imide, we are able to estimate the p*K*_b_ of **1** on the order of –20, in accord with such a moiety being a strong base and nucleophile as demonstrated through our reactivity.

### Complete N-atom transfer of the titanium nitride

**Scheme 3:**
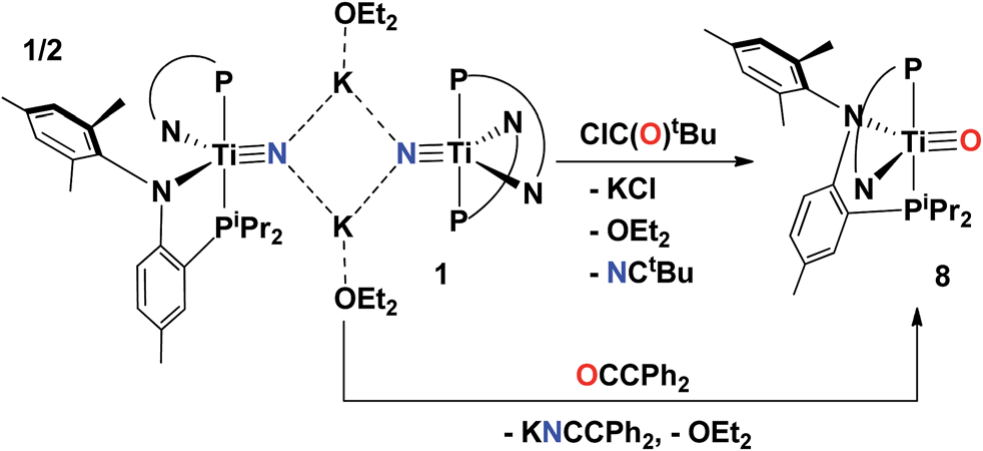
Salt metathesis, and N for O-atom transfer to form the oxo complex **8**.

A salt elimination reaction concurrent with complete N-atom transfer could be accomplished when complex **1** was treated with ClC(O)^t^Bu in toluene. Immediate formation of a purple solution is evidenced upon addition of the acid chloride, and workup of the reaction mixture followed by NMR spectroscopic characterization confirmed this purple species to be the oxo complex (PN)_2_Ti≡O (**8**) (Scheme 3) in near quantitative yield, ∼96%. Examination of the reaction mixture also revealed the formation of another product, which was identified as the organic nitrile NC^t^Bu based on NMR spectroscopic comparison to an authentic sample. The formation of the nitrile was monitored in ^1^H NMR spectroscopy to be formed in the same ratio as **8** *via* integration. A similar transformation involving N for O/Cl exchange was originally reported by Cummins using nitride complexes such as N≡W(N[^i^Pr]Ar)_3_ (Ar = 3,5-Me_2_C_6_H_3_) or the salt [Na][N≡Nb(N[Np]Ar)_3_] (Np = CH_2_^t^Bu).^31,86^ Likewise, Veige more recently reported N-atom transfer to various acid chlorides using {[^t^BuOCO]Mo≡N]Na(DMF)}_2_ [^t^BuOCO^3–^ = 2,6-C_6_H_3_(6-^t^BuC_6_H_3_O)_2_]. In their studies the authors were able to isolate the acylimido intermediate [^t^BuOCO]Mo≡NC(O)^t^Bu, and mechanistically probe for an azametallacylobutene species *en route* to N for O exchange.^87^

**Fig. 7:**
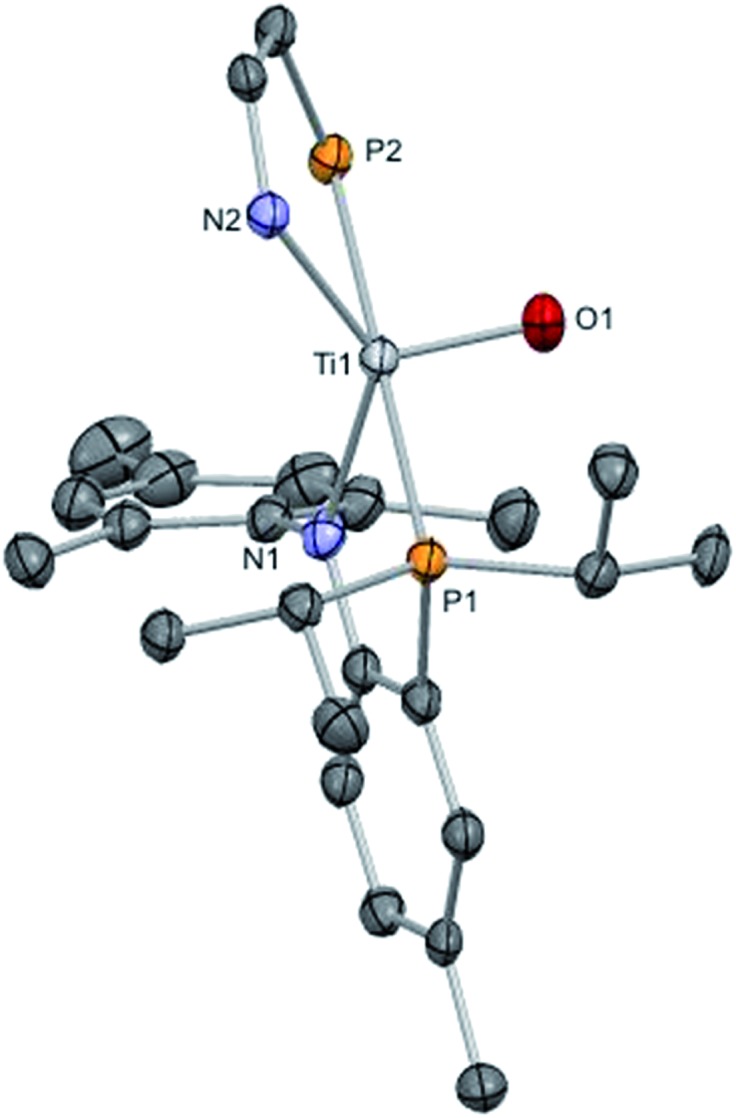
Solid state X-ray structure of complex **8**, depicting thermal ellipsoids at the 50% probability level. H atoms and the substituents of one depicted PN ligand have been omitted for clarity.

Complex **8** was characterized by ^1^H and ^31^P NMR spectroscopy and the latter displayed a sharp singlet at 13.3 ppm. Although there are no clear diagnostic signatures for **8** in the ^1^H NMR spectrum, monitoring the reaction mixture by ^1^H NMR spectroscopy revealed clean formation of a resonance at 0.8 ppm consistent with the pivaloyl nitrile (NC^t^Bu). Single crystal X-ray diffraction studies of **8** are in accord with a five-coordinate, mononuclear complex having a terminal oxo group, with a Ti≡O length of 1.644(2) Å (Fig. 7), well within the range of many reported terminal titanium oxo moieties.^58,88^ Rather unsurprisingly, the oxo complex closely parallels the molecular geometry of the parent imide, complex **7**, with a *τ*_5_ value of 0.769 and very similar P–Ti–P angle of 179.08(3)°. Furthermore, we explored the unusual purple color of this Ti(iv) complex *via* UV-vis analysis. The extremely low intensity absorption observed at 550 nm (*ε* = 174.8 M^–1^ cm^–1^) possibly corresponds to a similar phenomenon like that observed in MnO_4_^–^, where the complex absorbs yellow light to promote a LMCT band.

Complex **1** can also transfer the nitride atom to other carbonyl containing groups such as the ketene O=C=CPh_2_ (Scheme 3). Accordingly, addition of the ketene to **1** rapidly produces the signature purple color indicative in formation of **8**, as confirmed by both ^1^H and ^31^P NMR spectroscopy. However, upon addition of the ketene a salt is also produced which could be readily separated from the mixture *via* filtration. Given the insoluble nature of this yellow solid (presumably KN=C=CPh), we proceeded to treat it with Me_3_SiCl, immediately forming a salt (KCl) and a neutral species which was unequivocally identified to be the azaallene Me_3_SiN=C=CPh_2_ based on ^1^H NMR spectroscopic comparison to a literature report.^89^

### Solid state ^15^N NMR spectroscopic studies of titanium nitrides

**Fig. 8:**
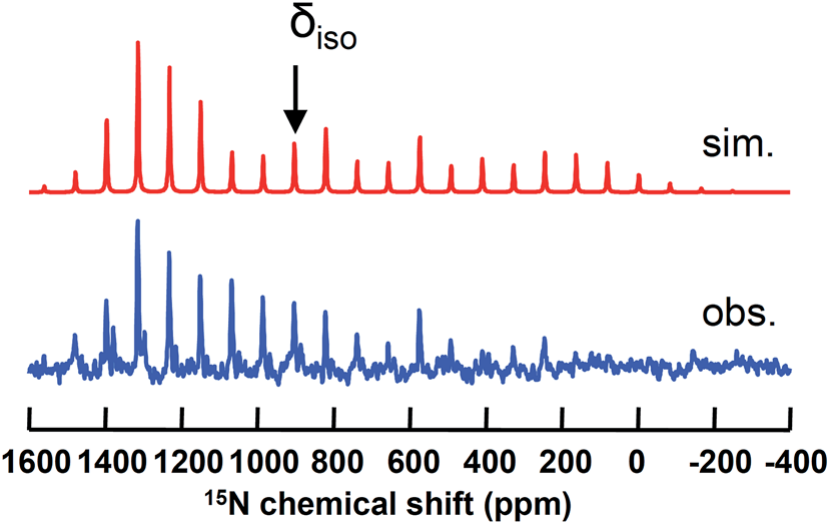
Simulated (top) and observed (bottom) solid state MAS ^15^N NMR spectrum of [K(2,2,2-Kryptofix)][(PN)_2_Ti≡ ^15^N].

Isolation of dinuclear and mononuclear nitrides of titanium presented us with a rare opportunity to investigate their axial symmetry. Hence, we prepared the 50% ^15^N enriched complex **1**-^15^N and converted it to the terminal nitride [K(2,2,2-Kryptofix)][(PN)_2_Ti≡ ^15^N] by addition of the cryptand (2,2,2-Kryptofix = 4,7,13,16,21,24-hexaoxa-1,10-diazabicyclo[8.8.8]hexacosane).^53^ We were able to analyze this discrete salt by magic angle spinning (MAS) ^15^N NMR spectroscopy. In accord with our expectations,^38,52^ this species reveals a significantly downfield chemical shift (*δ*_iso_ = 902 ppm), which is attributable to a highly deshielded terminal nitride species (Fig. 8). As presented in Fig. 8, the simulated ^15^N NMR solid state spectrum agrees well with experimentally collected values in both solid and solution state phases (*δ* = 958 ppm).^53^ Tensor components span a large chemical shift anisotropy (CSA) of nearly 1500 ppm, with *δ*_11_ ≈ *δ*_22_ = 1392 ± 50 and *δ*_33_ = –78 ± 50 ppm. Such anisotropy has been similarly observed in other mononuclear metal nitride species, namely ^15^N≡Mo(N[^t^Bu]Ar′)_3_ (Ar′ = 3,5-Me_2_C_6_H_3_) and [K(OEt_2_)]_2_[(^tBu^BDI)Ti≡ ^15^N(Ntolyl_2_)]_2_ (^tBu^BDI^–^ = [ArNC^t^Bu]_2_CH; Ar = 2,6-^i^Pr_2_C_6_H_3_), which also have a range of several hundred ppm for CSA in their tensor components.^52,90–92^ The observed ^15^N NMR chemical shift tensors were then used to compute the axial symmetry component, *κ* = 3(*δ*_22_ – *δ*_iso_)/(*δ*_11_ – *δ*_33_) = 1 ± 0.2. Gratifyingly, the *κ* value close to unity supports the triply bound nature of the Ti-nitride moiety in [K(2,2,2-Kryptofix)][(PN)_2_Ti≡ ^15^N], and which is the favorable bonding due to a pseudo-tetragonal environment shown in Fig. 3 (*vide supra*). The *κ* value observed here deviates notably from that reported previously for a less symmetrical Ti-nitride containing the BDI ligand, which has *κ* = 0.64.”.

### Computational studies scrutinizing the titanium nitride functionality

**Fig. 9:**
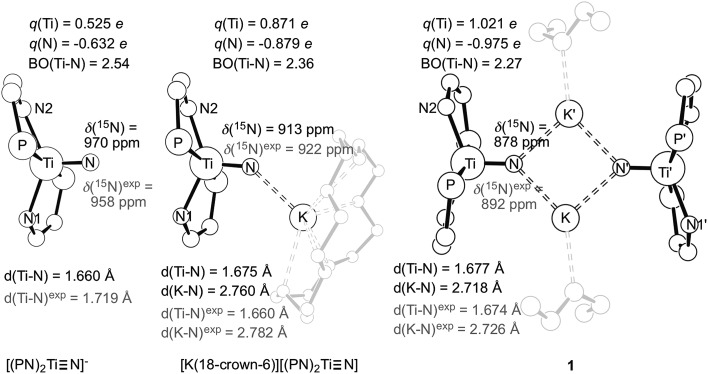
Equilibrium geometries for [(PN)_2_Ti≡N]^–^, [K(18-crown-6)][(PN)_2_Ti≡N] and **1** together with the most important structural metrics computed and determined by single crystal X-ray diffraction (grey). Also given are the computed and measured ^15^N NMR chemical shifts of the nitride centers as well as Mulliken charges of titanium, *q*(Ti), and the nitride center, *q*(N) together with the Mayer bond order (BO) index of the Ti≡N interaction.

To understand the observed characteristic reactivity and some of the measured physicochemical properties of the titanium nitride functionality we carried out a comprehensive computational and theoretical study on the putative anion [(PN)_2_Ti≡N]^–^ as well as complexes **1** and [K(18-crown-6)][(PN)_2_Ti≡N], that we recently characterized. Using the X-ray structures as guiding geometries, the slightly truncated models were fully optimized using the BLYP functional^93,94^ in combination with the Def2-TZVP(-f) basis set.^95^ The structural modifications included only the replacement of two *para*-methyl substituents to hydrogens in each PN^–^ ligand and were introduced to facilitate an all-electron DFT investigation for systems of the size of **1**, which still consists of 232 atoms after truncation. Dispersion has been also taken into account during optimizations using Grimme's D3 method.^96^ Dispersion turned out to be a critical component of our computational practice as it significantly contributed to finding accurate equilibrium geometries for both the dimer species, **1** and **2**, and for monometallic systems, that is [(PN)_2_Ti≡N]^–^, [K(18-crown-6)][(PN)_2_Ti≡N]. It is important to note that we observed a substantial improvement in the computed distances of weak Ti–P and K^+^···N interactions for the latter systems when compared to our earlier simulations without dispersion.^53^ As a matter of fact, the computational protocol outlined in the ESI† might offer useful practical solutions to few of the inborn weaknesses of standard DFT for such very extended transition-metal- and alkali-metal-containing systems. For example, one might obtain more realistic results for the notoriously overestimated bond lengths of weak metal–ligand interactions when taking into account inter ligand dispersion. One might be able to discard the surreal behavior of alkali-metal(s) in simulations, which was recently noted by Holland and co-workers to be detrimental for a systematic computational study on the alkali metal effect on Fe=N=N=Fe functionalities.^97^ As Fig. 9 reveals, the computed equilibrium structures are very similar to the experimentally determined molecular geometries established by single crystal X-ray diffraction studies. Also, in excellent agreement with our experiments, calculations predict the ^15^N NMR spectroscopic chemical shifts to be 913 ppm, 970 ppm and 878 ppm using the slightly truncated models of [(PN)_2_Ti≡N]^–^, [K(18-crown-6)][(PN)_2_Ti≡N], and **1**, respectively. These two benchmarks convincingly imply that our computer models capture the most salient features of the electronic structure of these molecular systems reasonably well and that the introduced structural simplification is acceptable. In particular, the agreement between the computed and experimentally observed effect of alkali-metal coordination on ^15^N NMR spectroscopic resonances supports the notion that the corresponding electronic structural changes are captured computationally in at least a plausible fashion.

**Fig. 10:**
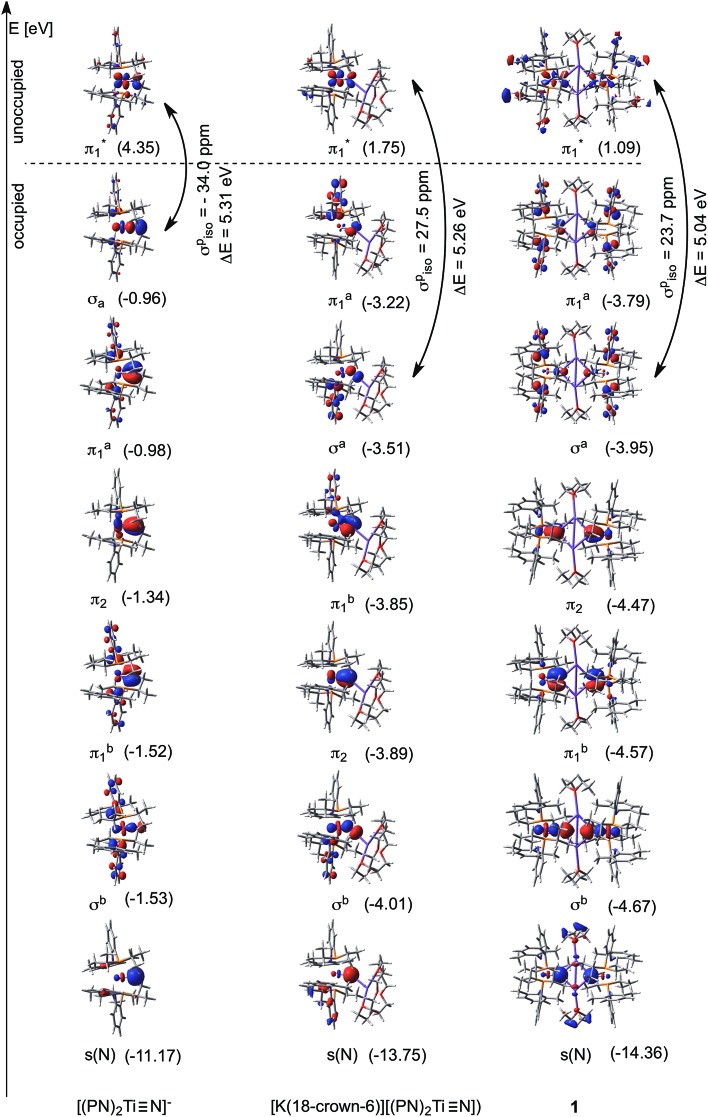
Most relevant occupied MOs for [(PN)_2_Ti≡N]^–^, [K(18-crown-6)][(PN)_2_Ti≡N] and **1** describing the electronic structure of the Ti≡N functionality. One of the unoccupied orbitals, 
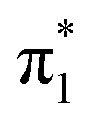
, which is critical for the paramagnetic shielding of N_nitride_ is also depicted.

As noted in our earlier communication,^53^ various computed electronic structure descriptors imply that the main difference in the Ti–N_nitride_ bonding is the different covalent/ionic character of the bond in the monomeric species [K(18-crown-6)][(PN)_2_Ti≡N] and [(PN)_2_Ti≡N]^–^. Namely, the Ti≡N bond in [(PN)_2_Ti≡N]^–^ exhibits a greater degree of covalent character than in [K(18-crown-6)][(PN)_2_Ti≡N]. In the case of the latter, the proximity of the K^+^ ion electrostatically stabilizes the highly charged nitride center and, concomitantly, induces a density shift from the Ti center towards the N_nitride_. This electron density shift induced by the proximity of K^+^ renders the titanium-nitride bond more ionic in [K(18-crown-6)][(PN)_2_Ti≡N] than in [(PN)_2_Ti≡N]^–^. As a matter of fact, the different ionic character can be clearly witnessed in the computed atomic charges of Ti (0.53 *e* in [(PN)_2_Ti≡N]^–^ and 0.87 e in [K(18-crown-6)][(PN)_2_Ti≡N]) and N (–0.63 *e* in [(PN)_2_Ti≡N]^–^ *vs.* –0.88 *e* in [K(18-crown-6)][(PN)_2_Ti≡N]) (Fig. 10). The reduced bond order in [K(18-crown-6)][(PN)_2_Ti≡N] (2.36 *vs.* 2.54 in [(PN)_2_Ti≡N]^–^) also conforms to a lower degree of covalent character of the Ti≡N bond in this structure. These pronounced differences in Ti–N_nitride_ bonding are further enhanced with the presence of two potassium ions in **1**. In particular, atomic charges (*q*(Ti) = 1.02 *e* and *q*(N) = –0.975 *e*) as well as the reduced bond order of 2.27 indicate the higher ionic and lower covalent character of the Ti≡N bond in **1** to either [K(18-crown-6)][(PN)_2_Ti≡N] or [(PN)_2_Ti≡N]^–^ (Fig. 9).

More thorough insights into the electronic structure changes induced by the alkali-metal ion are gained through a close inspection of the molecular orbitals (MOs) that describe the bonding of the titanium-nitride functionality. A conceptual MO-diagram that highlights the most relevant molecular orbitals of the metal–nitride interactions is given in Fig. 3, implying one σ- and two π-bonds and a lone pair at N_nitride_. Instead of the expected four MOs representing these functions, however, Fig. 10 depicts six occupied orbitals for each Ti≡N functionality merely because the σ-bond as well as one of the Ti–N π-bonds can be defined by two MOs. In detail, the Ti–N_nitride_ σ-interaction of each Ti≡N functionality in Fig. 10 is represented by two MOs, σ^b^ and σ^a^, which evolve due to the formation of a bonding (b) and an anti-bonding (a) combination with a π-type orbital of the PN^–^ ligand(s). Similarly, orbitals πb1 and πa1, which appear again as a bonding/anti-bonding pair with a PN^–^ ligand orbital, together characterize one of the Ti–N_nitride_ π-interactions. In addition, π_2_ represents the other π-type interaction which lies about in the P–Ti–P plane whereas, as hypothesized in Fig. 3, the lone pair at N_nitride_ has a strong component of s atomic orbital character and it is of low energy.

Although the crown ether-encapsulated K^+^ breaks the symmetry in [K(18-crown-6)][(PN)_2_Ti≡N] as well as it alters the ordering of the orbitals, the computed MOs characterize the same Ti–N_nitride_ interactions as for [(PN)_2_Ti≡N]^–^. Moreover, the analogous MOs can also be intuitively recognized for the dimer species **1** (Fig. 10), for which only the symmetric combinations are illustrated for clarity. The side-by-side comparison of the corresponding MOs reveal that the σ-interaction, mostly σ^a^, as well as πa1 experiences a significant change in character when being confronted by the alkali metal ion(s); the atomic contribution of Ti decreases whereas that of N_nitride_ increases in these MOs when going from [(PN)_2_Ti≡N]^–^ to [K(18-crown-6)][(PN)_2_Ti≡N] or **1**. This difference in corresponding MOs conforms to the additional polarization of the Ti–N_nitride_ bond that was discussed above and which was supported with charge distribution measures and bond descriptors. Finally, the stabilization effect of the nearby cation on the electron rich N_nitride_ can be clearly witnessed in the computed orbital energies, which are much more negative in **1** and in [K(18-crown-6)][(PN)_2_Ti≡N] than in [(PN)_2_Ti≡N]^–^.

The observed and computed ^15^N NMR chemical shift of N_nitride_ is not only useful for precisely determining the identity of these species in solution, but can also be utilized as a diagnostic tool that gives direct information on the chemical environment about N_nitride_ and, as such, on the Ti–N_nitride_ interaction. In particular, the gradual upfield shift of 50–30 ppm when going from [(PN)_2_Ti≡N]^–^ to [K(18-crown-6)][(PN)_2_Ti≡N] and **1** suggests a subtle change in the electronic structure due to the nearby cation(s). In order to put this trend of ^15^N_nitride_ chemical shift into context with the underlying electronic structures it is critical to realize that magnetic shielding of heavy nuclei does not directly correlate with the electron density at the nucleus. Rather, heavy nuclei, such as ^15^N, have access to low-energy molecular orbitals of p and d-type atomic contributions that make the electron density around these nuclei very dynamic in the sense that local fluctuations of the electron cloud become more prevalent in an external magnetic field. The magnetic shielding that results from these electron density fluctuations is conventionally referred to as paramagnetic shielding, σ^p^, and is typically more sensitive to the changes in chemical bonding than the diamagnetic shielding, σ^d^, which originates from tightly-bound electron density at the nucleus. In a recent study we discussed the basic relationships of such density fluctuations with MOs, shielding tensors and chemical shifts and, by scrutinizing vanadium- and molybdenum-*cyclo*-P_3_ complexes, we showed how a thorough analysis of shielding tensors of heavy nuclei can lead to a conceptual understanding of the bonding characteristics of unusual transition metal–ligand interactions.^98^

To understand what makes the N_nitride_ nucleus more shielded in the case of K^+^ ligated species, we computed and analyzed the ^15^N magnetic shielding tensors of the above-described slightly truncated versions of the complexes [(PN)_2_Ti≡N]^–^, [K(18-crown-6)][(PN)_2_Ti≡N] and **1**. In general, the magnetic shielding at a NMR-active nucleus and the resulting chemical shift can be calculated from first principles to a reasonable degree of accuracy^91,99–101^ and, accordingly, the computed values shown in Fig. 10 also agree well with measured solution-state ^15^N NMR spectroscopic resonances. In addition, the above-discussed large chemical shift anisotropy (CSA) is reproduced computationally (∼1500 ppm *vs.* 1470 ppm) for [(PN)_2_Ti≡N]^–^ as well as the corresponding principal components of the shielding tensor (*δ*_11_ = 1562.2 ppm, *δ*_22_ = 1285.0 ppm and *δ*_33_ = 61.9 ppm) are in line with the experimentally determined components in solid state (*δ*_11_ ≈ *δ*_22_ = 1392 ± 50 ppm and *δ*_33_ = –78 ± 50 ppm) for [K(2,2,2-Kryptofix)][(PN)_2_Ti≡N]. Due to omitting the K(2,2,2-Kryptofix)^+^ counter ion and other condensed phase effects in the simulations, however, a difference of about 280 ppm between *δ*_11_ and *δ*_22_ appears *in silico*, which manifests in an underestimated axial symmetry component (*κ* = 0.63 *vs.* app. 1) for the computationally considered model system, [(PN)_2_Ti≡N]^–^. As expected, the diamagnetic term displays a narrow range of shielding contributions with computed σ^d^ values of 339.7, 341.3 and 347.7 ppm for ^15^N_nitride_ in [(PN)_2_Ti≡N]^–^, [K(18-crown-6)][(PN)_2_Ti≡N] and **1**, respectively. The paramagnetic contribution, on the other hand, differs significantly with computed shielding parameters of –1050.3, –996.3 and –967.3 ppm, respectively. These values illustrate the general concept mentioned above that σ^p^ dominates the overall shielding of heavy nuclei and, thus, determines the chemical shift *δ*_avg_ against a reference.

As we demonstrated recently,^98^ the thorough analysis of the paramagnetic contribution to the shielding can be challenging, because one has to decompose the sum of a great number of individual magnetic fields originating from occupied–unoccupied orbital pairs that couple through the angular momentum operator. In addition, the magnetic fields generated by what is most spontaneously understood as electron density fluctuations are hard to visualize or imagine. Finding a few numbers of distinguishing orbital-pairs that make a decisive contribution and that are characteristic for the certain species under investigation is the key to understanding such paramagnetic shielding. Nevertheless, we could pinpoint one occupied orbital, σ^a^, whose contribution to shielding changes significantly in the presence of a nearby K^+^ (Fig. 10). For example, the total paramagnetic shielding contribution of σ^a^ is –504.6 ppm in [(PN)_2_Ti≡N]^–^ whereas it drops to –128.0 ppm in [K(18-crown-6)][(PN)_2_Ti≡N] and even becomes shielding (157.6 ppm) in **1**. This σ^a^ MO couples most intensively with the vacant π* orbitals corresponding to the antibonding combinations of Ti–N_nitride_ π-bonds, *e.g.* 
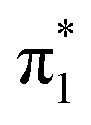
 illustrated in Fig. 10. In this context it is worth noting that Morokuma, Schrock, Griffin and Cummins reported very similar findings for the unusual ^31^P NMR chemical shielding tensors of terminal phosphide (M≡P) complexes of molybdenum and tungsten.^91^ In the latter systems the σ(M≡P) and π*(M≡P) MO mixing makes the primary contribution to the ^31^P paramagnetic shielding due to the components of the applied magnetic field which are oriented perpendicular to the M≡P bond. Fig. 10 also illustrates how the paramagnetic shielding contribution of σ^a^ ↔ 
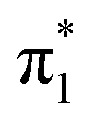
 mixing varies for each nitride species. The coupling of these orbitals in an external magnetic field induces a density flow that has a deshielding effect in [(PN)_2_Ti≡N]^–^, whereas it generates a shielding effect in [K(18-crown-6)][(PN)_2_Ti≡N] and **1**, where the K^+^ affects the bonding characteristics. The energy gap (Δ*E*) between interacting orbitals is also given in Fig. 10, as the coupling efficiency is inversely proportional to the energy difference between the interacting orbitals.^99^ For example, the ^31^P NMR chemical shift of triple bonded phosphorous has been found to correlate very well with the σ–π* energy gap as well as we also reported the significance of energy gap of coupling orbitals in determining the chemical shielding of phosphorous nuclei in metal-*cyclo*-P_3_ complexes.^98^ The very small differences in energy gaps (Δ*E* in Fig. 10), however, cannot account for the qualitatively different paramagnetic shielding effects of σ–π* coupling in the studied systems. Rather, the qualitative difference of paramagnetic contributions, *i.e.* deshielding in [(PN)_2_Ti≡N]^–^ and more shielding in K^+^–ligated systems, implies that the spatial distribution of these molecular orbitals alters upon K^+^ coordination. Accordingly, the localization of σ^a^ on the nitride center as well as the localization of 
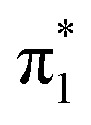
 on Ti in K^+^–ligated systems results in a constructive orbital overlap when these orbitals couple through the angular momentum operator, in contrast to the destructive overlap (deshielding effect) in naked [(PN)_2_Ti≡N]^–^. Hence, the upfield shift of ^15^N_nitride_ resonance is linked to the K^+^ induced electronic structure change of the titanium-nitride functionality as demonstrated through a quantum chemical analysis of the corresponding shielding tensors.

Finally, our calculations reveal, in line with the N···K distances larger than 2.7 Å, that the Ti≡N···K^+^ interaction is merely electrostatic in nature without any sign of covalent contribution. This electrostatic interaction, beyond inducing the above-scrutinized change of the Ti≡N functionality, represents the main source of thermodynamic stabilization and driving force for forming well-structured complexes such as [K(18-crown-6)][(PN)_2_Ti≡N] and the dimer **1**.

## Conclusions

No previous study has thoroughly explored or elucidated the reactivity of molecular titanium nitrides prior to the work presented herein. Our findings imply in general that the Ti≡N functionality is a very strong nucleophile, which readily reacts with electrophilic reagents to serve as a full or partial N-atom transfer reagent. A combined experimental and theoretical investigation has helped us understand the bonding and structure in these systems as well as rationalize the nature of the ^15^N chemical shift of the nitride ligand and the role of the K^+^ counteraction. Looking forward, we seek to further understand how this nitride moiety can be tuned and modified to generate ammonia under hydrogen, and further, generate the nitride instead from nitrogen as opposed to azide to extend the scope of this chemistry to a more sustainable and atom-efficient process.
