# Supplementary material for: Molecular titanium nitrides: nucleophiles unleashed
Source: Chem Sci. 2016 Sep 22;8(2):1209–24. doi: 10.1039/c6sc03422e (PMC5369542; doi:10.1039/c6sc03422e)
Supplement: Supplementary file 1 [file SC-008-C6SC03422E-s001.pdf]

Supporting Information for:
Molecular Titanium Nitrides: Nucleophiles Unleashed

Lauren N. Grant,[§] Balazs Pinter,[ǀ] Takashi Kurogi,[§] Maria E. Carroll,[§] Gang Wu,[‡] Brian C. Manor,[§] Patrick J. Carroll[§] and Daniel J. Mindiola[§,*]

[§]*Department of Chemistry, University of Pennsylvania, 231 South 34th Street, Philadelphia PA, 19104.*
[ǀ]*Eenheid Algemene Chemie (ALGC), Vrije Universiteit Brussel (VUB), Pleinlaan 2, 1050, Brussels (Belgium).*
[‡]*Department of Chemistry, Queen's University, Kingston, Ontario, Canada K7L 3N6.*

Corresponding author: E-mail, mindiola@sas.upenn.edu

Table of Contents

A. NMR Data S2-S9

B. Phosphonylimide LT NMR experiments S10-S11

C. Computational Details S11-S17

D. Crystallography Details S16-S17

E. Solid state ^{15}N NMR Details S18

F. References S19-S20

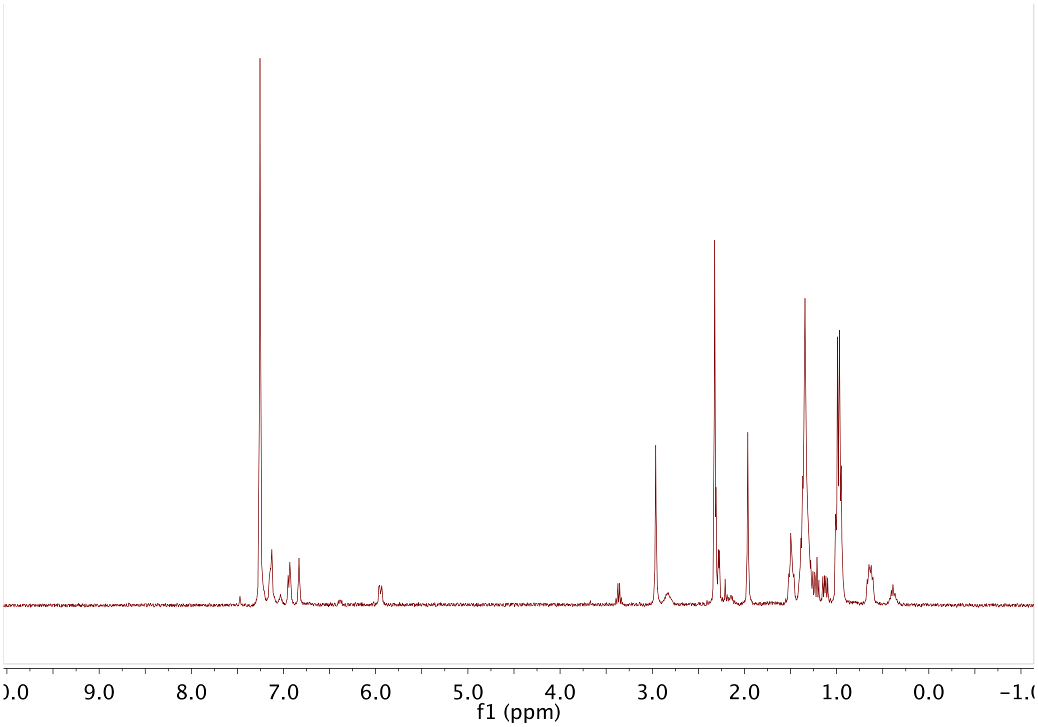

Figure S1: ^{1}H NMR Spectrum of **2** in C_6D_6, 400 MHz, 300K

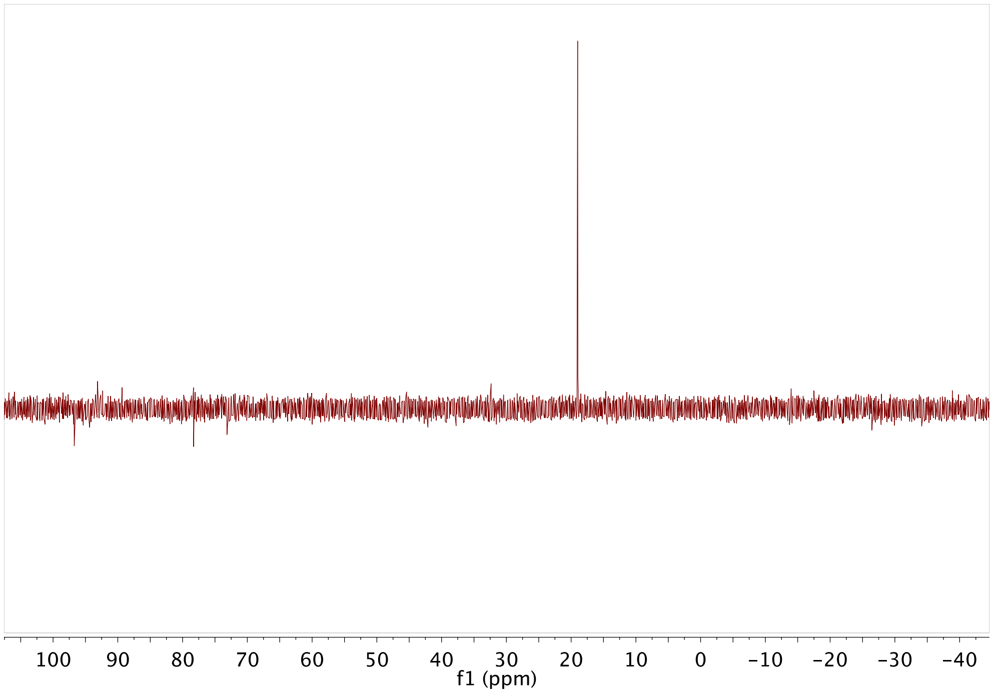

Figure S2: $^{31}P\{^{1}H\}$ NMR Spectrum of **2** in C_6D_6, 162 MHz, 300K

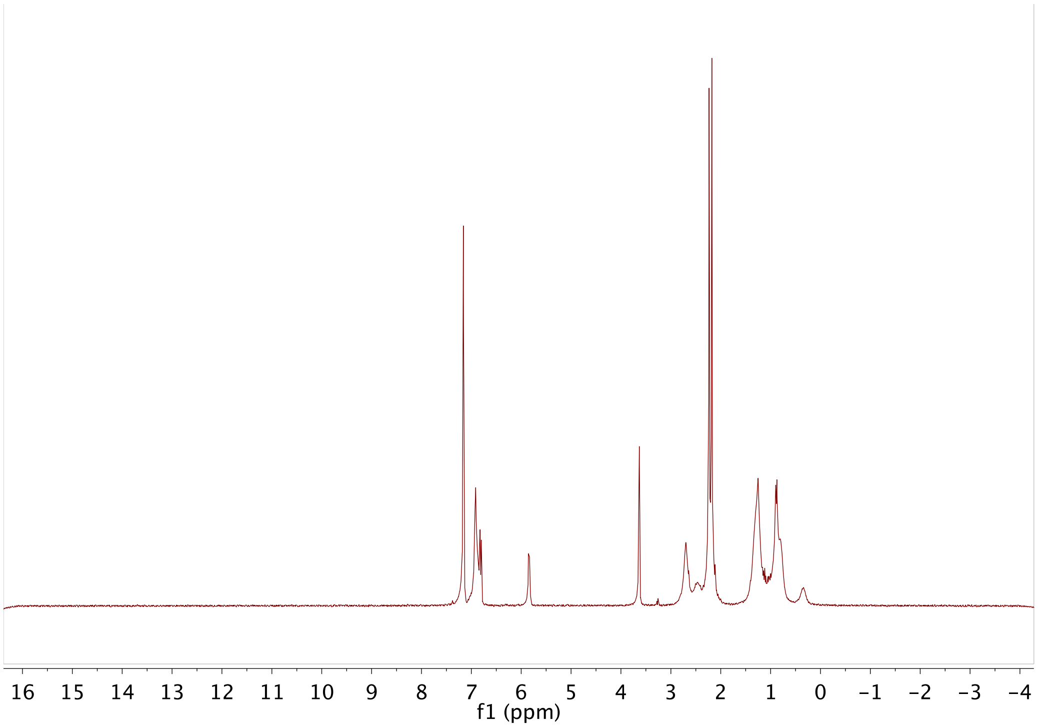

Figure S3: ^{1}H NMR Spectrum of **3** in C_6D_6, 400 MHz, 300K

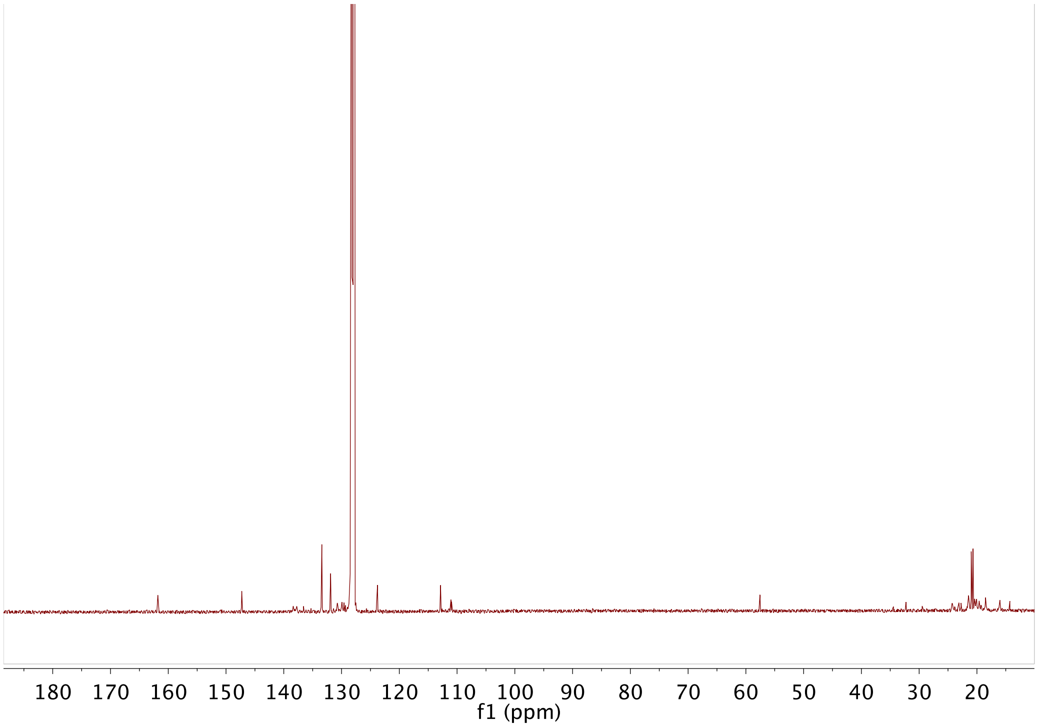

Figure S4: $^{13}C\{^{1}H\}$ NMR Spectrum of **3** in C_6D_6, 125.8 MHz, 300K

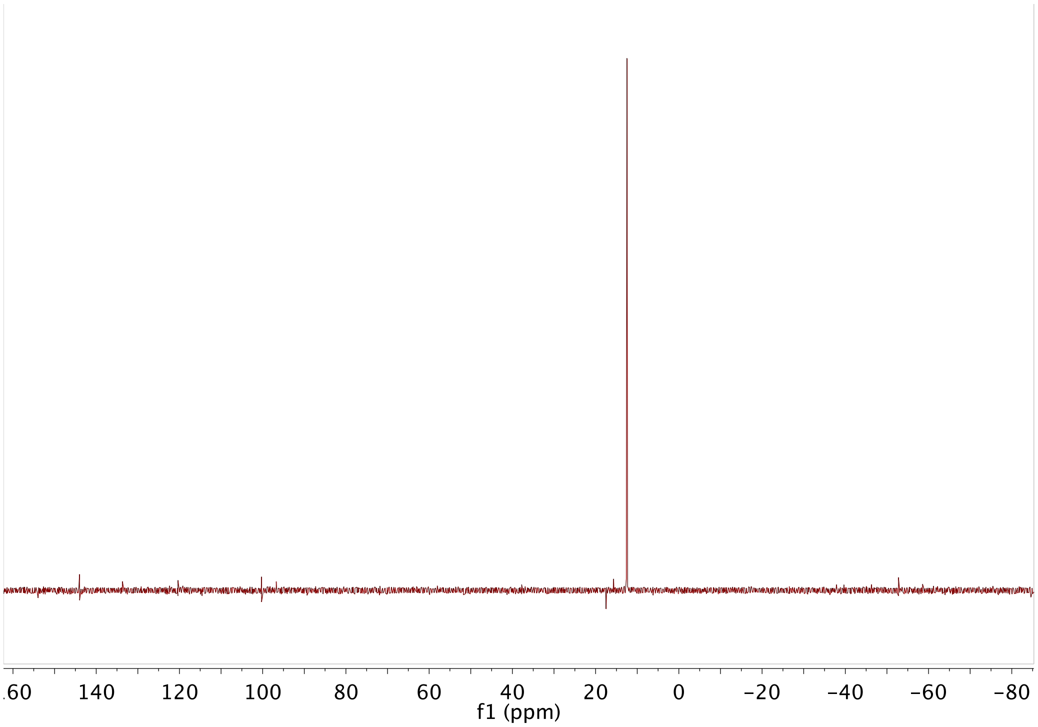

Figure S5: $^{31}P\{^{1}H\}$ NMR Spectrum of **3** in C_6D_6, 162 MHz, 300K

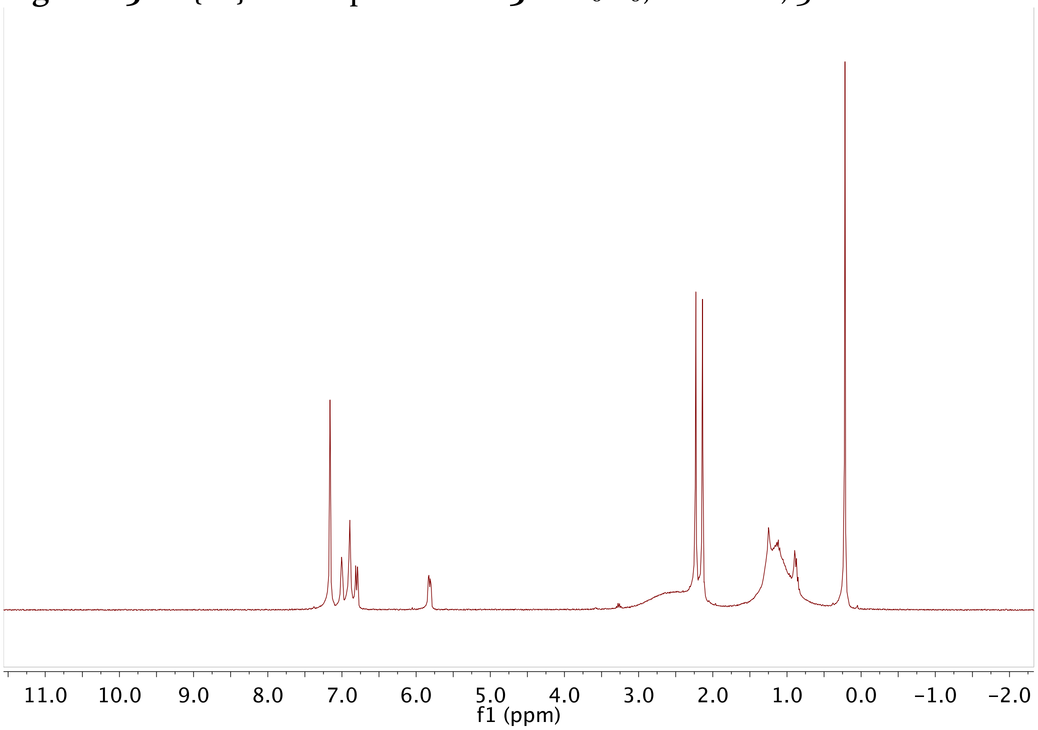

Figure S6: ^{1}H NMR Spectrum of **4** in C_6D_6, 400 MHz, 300K

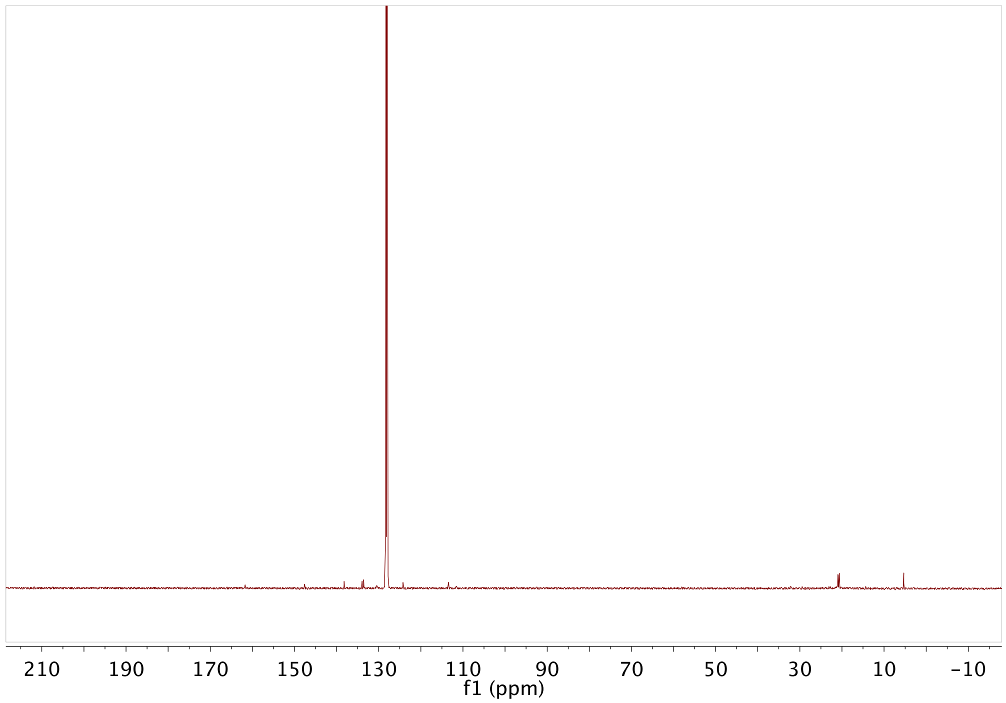

Figure S7: $^{13}C\{^{1}H\}$ NMR Spectrum of **4** in C_6D_6, 125.8 MHz, 300K

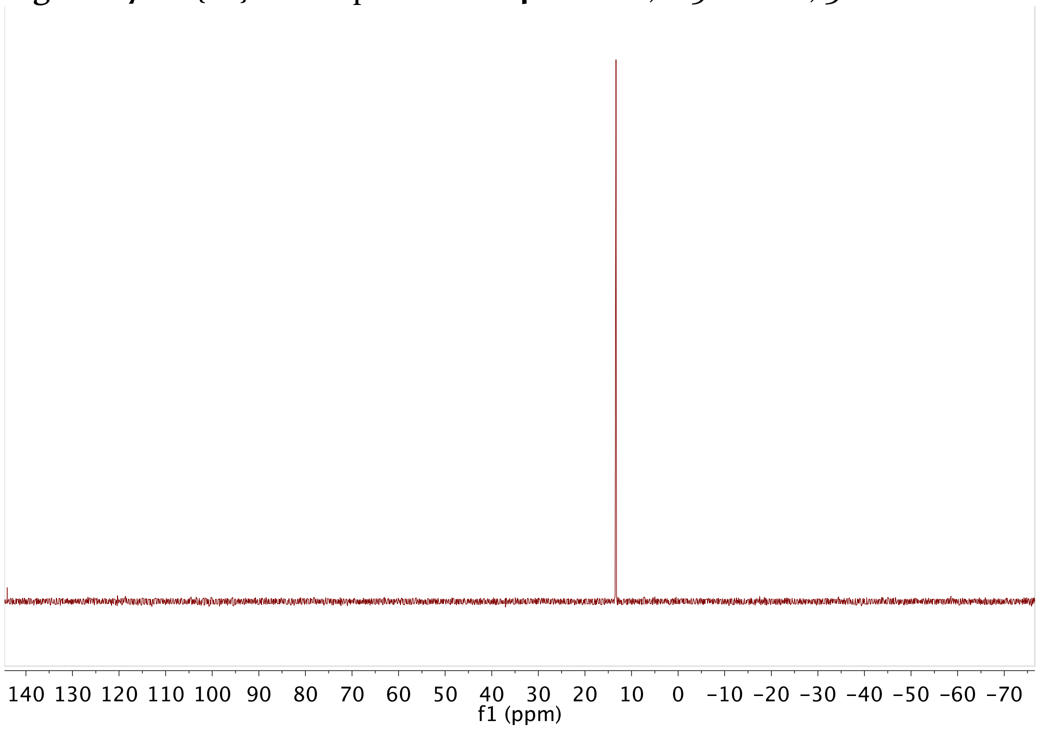

Figure S8: $^{31}P\{^{1}H\}$ NMR Spectrum of **4** in C_6D_6, 162 MHz, 300K

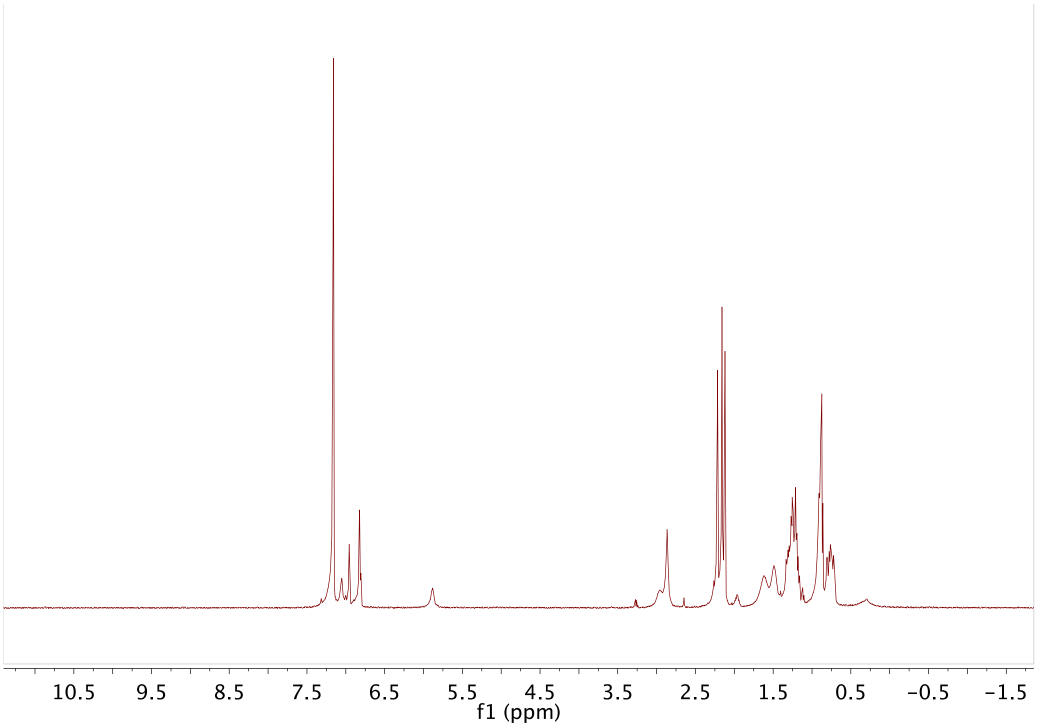

Figure S9: ^{1}H NMR Spectrum of **5** in C_6D_6, 400 MHz, 300K

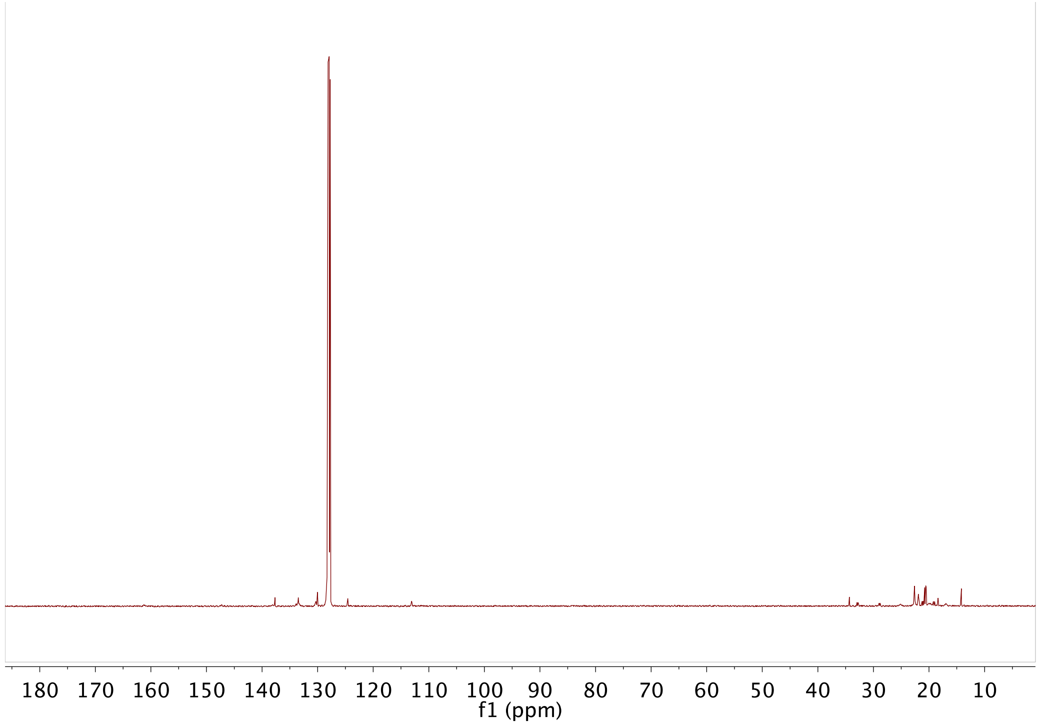

Figure S10: $^{13}C\{^{1}H\}$ NMR Spectrum of **5** in C_6D_6, 125.8 MHz, 300K

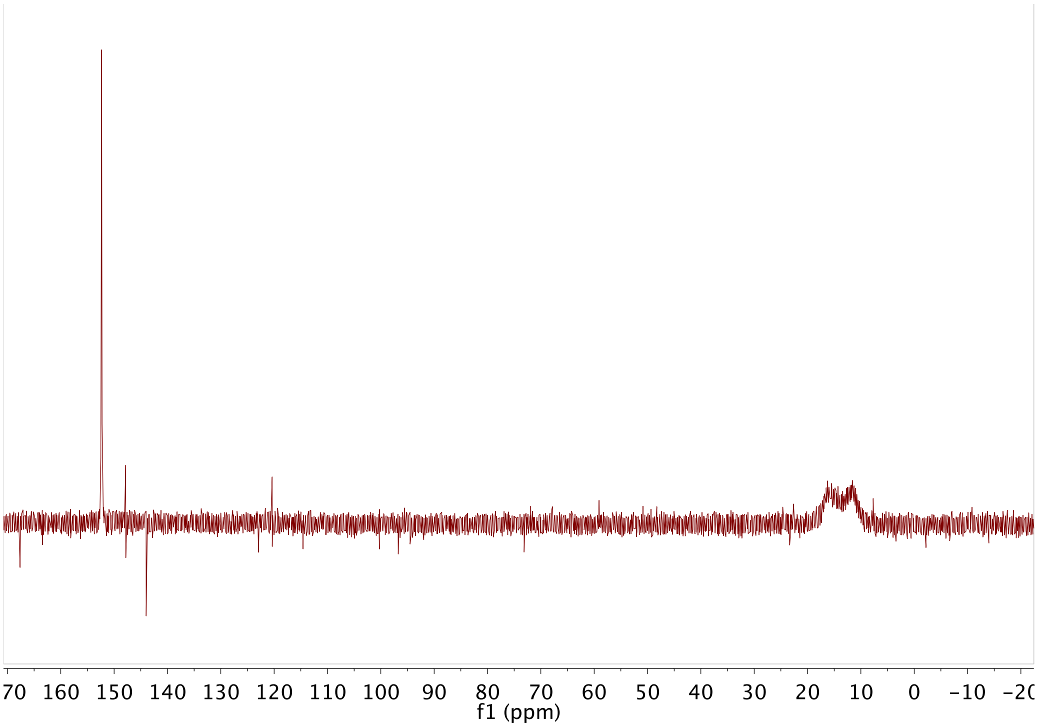

Figure S11: $^{31}P\{^1H\}$ NMR Spectrum of **5** in C_6D_6, 162 MHz, 300K

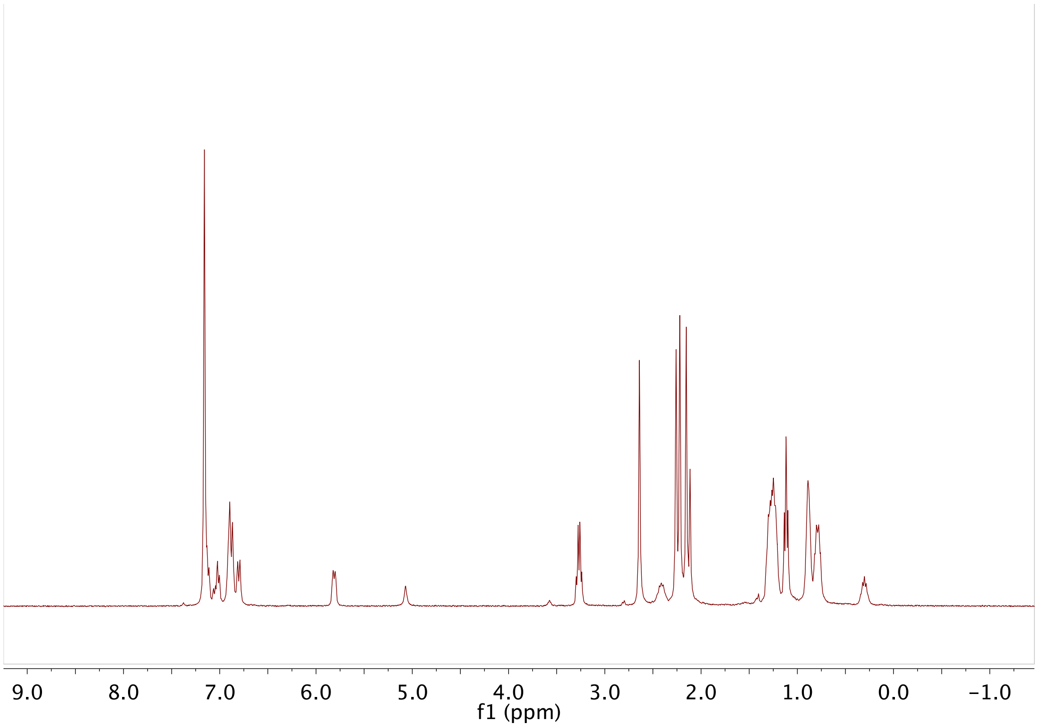

Figure S12: ^1H NMR Spectrum of **7** in C_6D_6, 400 MHz, 300K

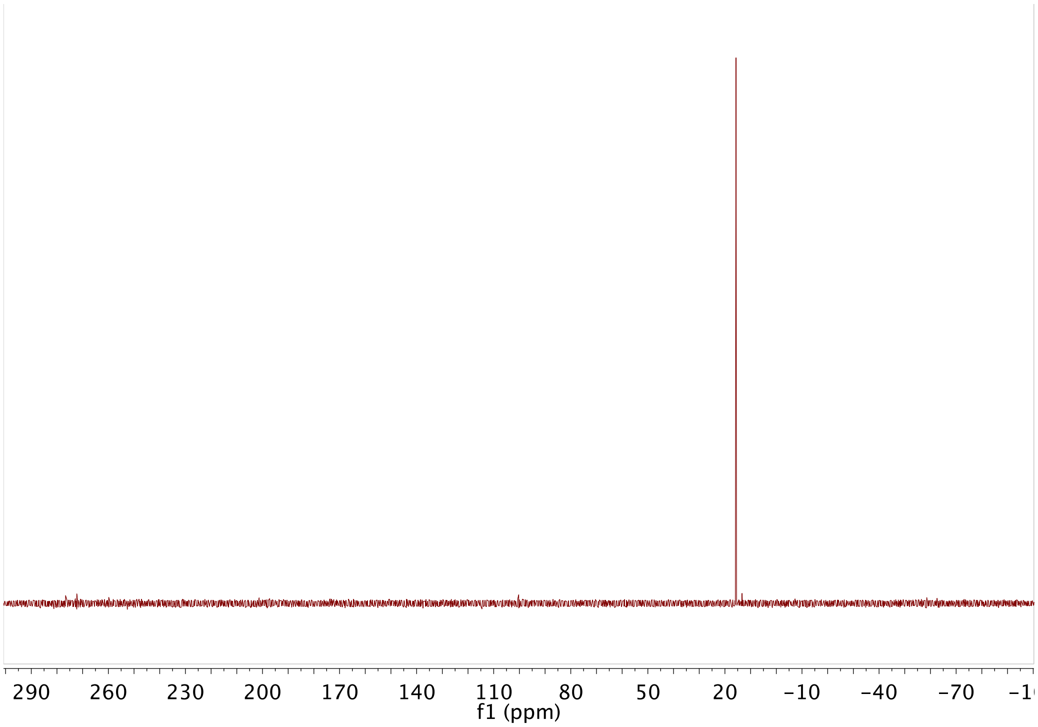

Figure S13: $^{31}P\{^{1}H\}$ NMR Spectrum of **7** in C_6D_6, 162 MHz, 300K

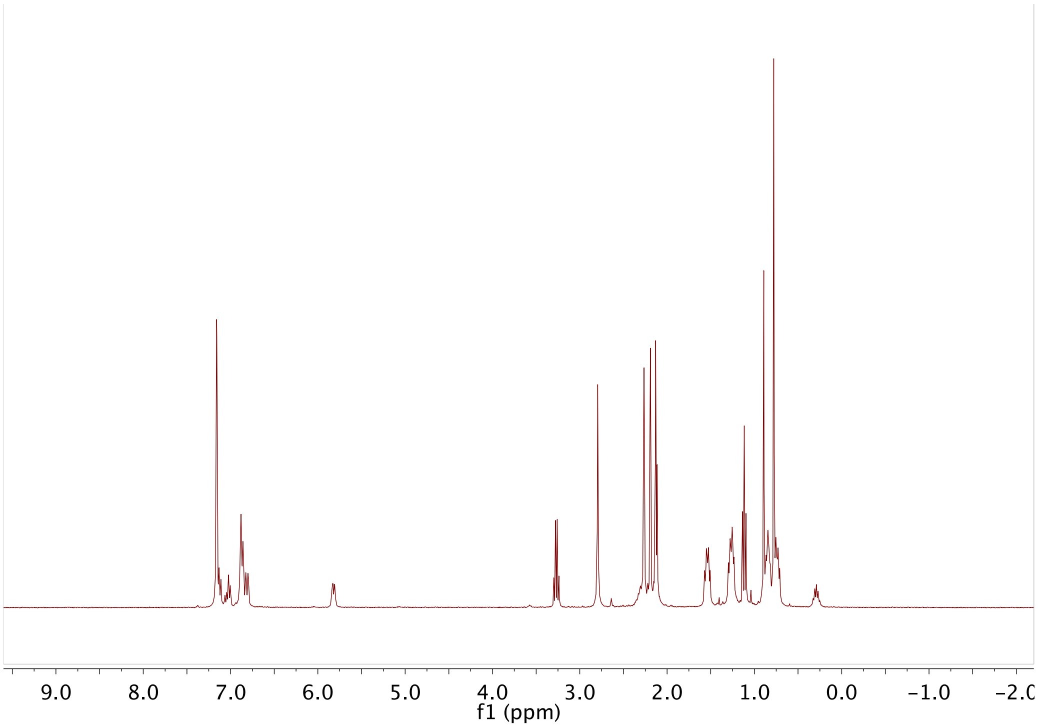

Figure S14: ^{1}H NMR Spectrum of **8** in C_6D_6, 400 MHz, 300K

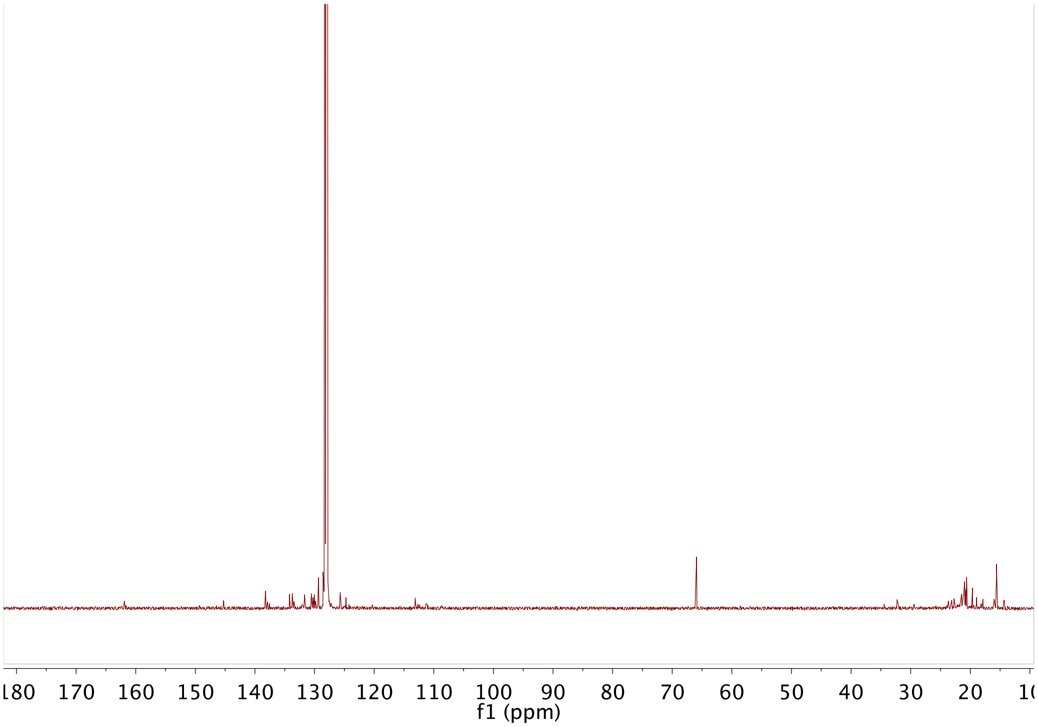

Figure S15: $^{13}C\{^{1}H\}$ NMR Spectrum of **8** in C_6D_6, 125.8 MHz, 300K

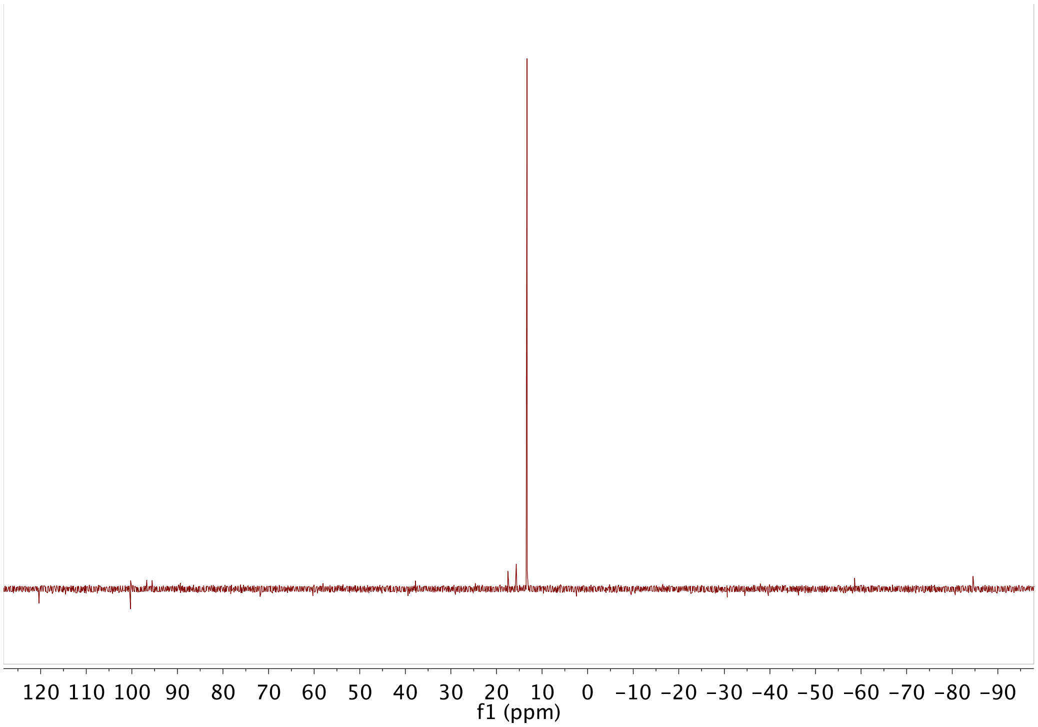

Figure S16: $^{31}P\{^{1}H\}$ NMR Spectrum of **8** in C_6D_6, 162 MHz, 300K

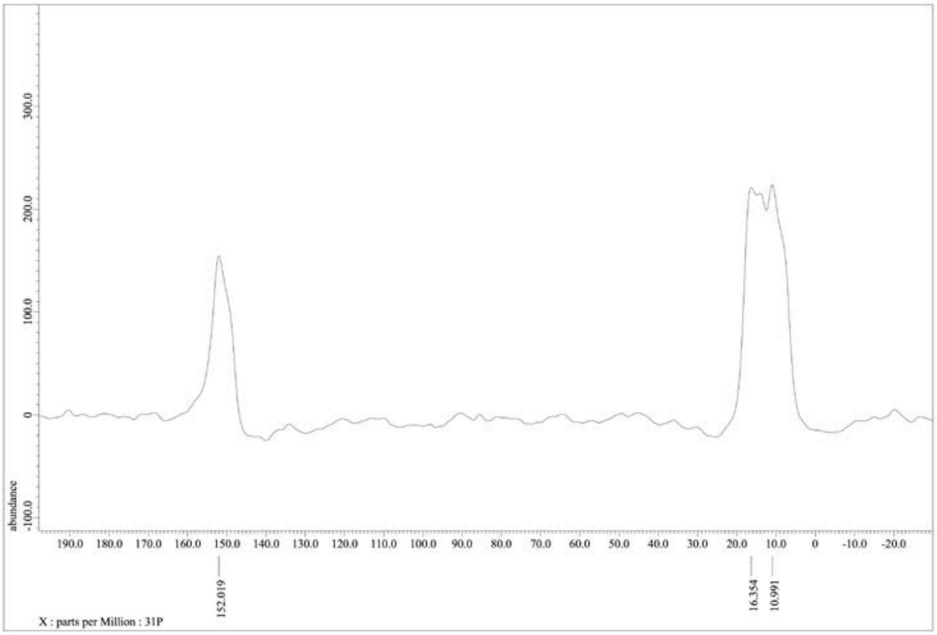

Figure S17: ^{31}P NMR Spectrum of **5** in C_7D_8, 162 MHz, 265K

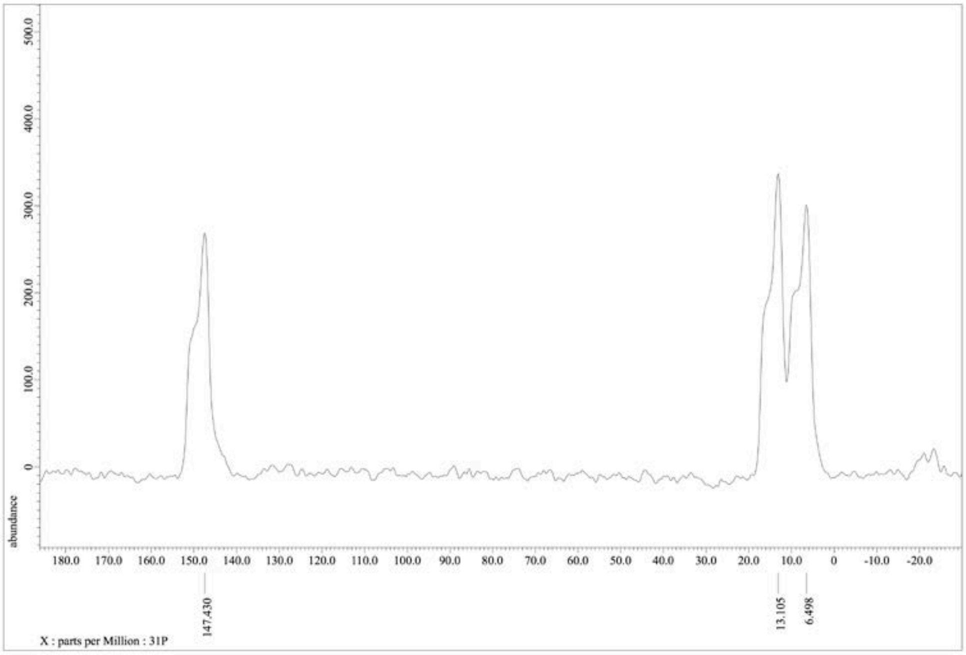

Figure S18: ^{31}P NMR Spectrum of **5** in C_7D_8, 162 MHz, 219K

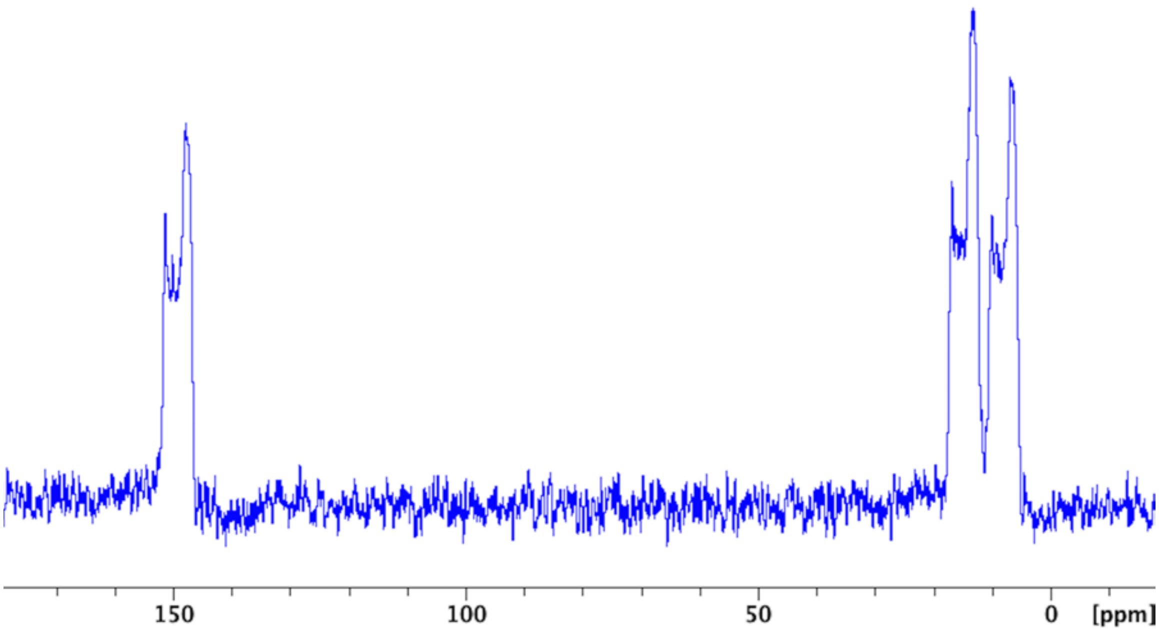

Figure S19. $^{31}P\{^{1}H\}$ NMR spectrum of **5**, taken at 219 K in C_7D_8, 162 MHz.

Computational Details. All calculations were carried out using DFT as implemented in the ORCA program package[1]. Final geometry optimizations were performed using the BLYP[2,3] functional and the all-electron def2-TZVP(-f)-ZORA[4] basis set in combination with the auxiliary basis set def2-TZV/J[5]. To accelerate geometry optimizations we used the resolution of the identity, R[6], approximation. For these optimizations a tight convergence of the wavefuntion was demanded on grid quality of Grid4 (also using SlowConv). The scalar relativistic zero'th order regular approximation (ZORA)[7] was employed to take into account relativistic effects whereas to dispersion was considered using Grimme's D3 method in all ORCA calculations. Our experience with the optimizations of the investigated extended molecules (~ 200 atoms) is that the convergence to the equilibrium structure is much faster when optimizing in Cartesian coordinates (COPT). Finally, the above-described final geometry optimizations were started from pre-optimized structures obtained via a lower level of theory (BLYP/def2-SV(P), def2-SVP/J, ZORA, RI, d3, LooseSCF, Grid3).

We used the NMR module of ADF2013[8] code to compute ^{15}N NMR chemical shifts on the equilibrium structures obtained via the protocol described above. The functional employed consisted of the local density approximation of Vosko, Wilk, and Nusair (LDA VWN)[9] augmented with the nonlocal gradient correction PW91

from Perdew and Wang[10]. This functional has been shown to provide reliable chemical shift values even for heavy atom containing transition metal complexes[11]. The full electron basis set TZVP was utilized in these calculations and relativistic corrections were applied using ZORA. The computed isotropic shielding values of complexes were referenced to that of ammonia modeled in the same way.

Cartesian coordinates of the optimized structures. Values are in Å.

$[(PN)_2Ti{\equiv}N]^-$

Ti	13.62669808760018	3.96420658351770	11.08650793112511
P	13.38157696391465	2.75197968063771	13.42612122613409
P	13.89249524480639	5.08840759579496	8.70385519971816
N	11.83642381437701	2.68945822638232	10.88118357682088
N	15.77143680538267	3.76708713018363	10.59956338697567
N	13.27518608595241	5.43206415827361	11.77695419935315
C	11.16037577514131	2.06506699078003	11.89920172070712
C	9.88654539474408	1.43640096805512	11.72404261816514
H	9.43105220533442	1.44354201242791	10.73716982490926
C	9.21361596283514	0.83918815059931	12.78434842156115
H	8.24090453866257	0.37973587154015	12.60216359212469
C	11.00944062052555	1.42532035963551	14.27100128579559
H	11.43744664258646	1.44001172397940	15.27175747054117
C	11.71966257337982	2.01851259000642	13.22066915149744
C	13.18684887185236	3.81738699407600	14.96422360066922
H	12.89396212419045	3.13921273430080	15.77894366292507
C	12.07516269320404	4.86088176058260	14.75797368919601
H	12.30371259741699	5.48301142602752	13.88165947260527
H	11.10343995972214	4.38089088270874	14.59495250060202
H	12.00116031943368	5.49917747295321	15.65232601458707
C	14.51975417445203	4.49374630327733	15.32183500475256
H	14.42401093525893	5.03385468919605	16.27564744398813
H	15.34483894863827	3.77744397570099	15.41771920675484
H	14.78554311461530	5.21705954422903	14.54551718002616
C	14.46564553485123	1.30311064464931	13.97199142542481
H	15.48196542287976	1.68876517633844	13.84264066305601
C	14.31889717590508	0.81555912760085	15.42113765561150
H	15.05178238095500	0.01785586379244	15.61793888433257
H	14.50108280365205	1.61564738421647	16.14762737886045
H	13.32067591050793	0.40053307231526	15.60636218279475
C	11.17496521514937	2.76264141982546	9.62759462019936
C	11.40250192613497	1.76546896485746	8.64950441195957
C	10.76580848777050	1.86624010136729	7.40494613917865
H	10.95278731900272	1.10331358280185	6.64840042741855
C	9.91215478827124	2.93757022316057	7.12284667232013
C	9.66932046854489	3.90628496967187	8.10291674820671
H	8.99458600782016	4.73684035035855	7.89227342017882
C	10.27328088546835	3.82510596239616	9.36584039663126
C	12.30266112348611	0.59746715891181	8.97500596705930
H	12.45578492957968	-0.03792735588797	8.09401649374747
H	13.27992043172245	0.93770270626407	9.33815672520149
H	11.87422970338450	-0.01461704899741	9.78088007568347
C	9.94572022736502	4.80651127716216	10.46423372754598
H	9.36565896427783	5.65155034001391	10.07389460656810
H	9.35188171303673	4.31065337223033	11.24588855098617
H	10.85611977755755	5.17672898417887	10.95835792781692
C	16.44012290593769	4.40017686729640	9.58223208422858
C	17.86554666558206	4.38486538611650	9.45230273175799
H	18.45191166142771	3.84013304621534	10.18775267861740
C	18.51387938411504	5.06278132361297	8.42577611498193
H	19.60308179274010	5.02641623100471	8.37303565427240
C	17.79361712138070	5.79812398318866	7.47103359254700
C	16.39820896309266	5.81698039905980	7.56994429378301
H	15.82606317929166	6.39670889390594	6.84791056651523
C	15.71547781981061	5.12928596463152	8.57998482721499
C	13.32887519234404	4.28125703770986	7.08936962208721
H	12.28580477097369	4.01970694345602	7.29336194626218
C	14.12366869521213	2.97786197576910	6.89924222332217
H	15.15478238716385	3.19386650420992	6.59457412904897
H	14.16747803219602	2.39358643632887	7.82368732175463
H	13.64989552424189	2.35341474130950	6.12889921753835
C	13.36405446611479	5.13980773111330	5.81569764713722
H	12.94905505120738	4.56512273746632	4.97325991395446
H	12.76680658783917	6.05339847903101	5.91571928112138
H	14.38920754231016	5.42408179760598	5.54884352200060
C	13.41701561542652	6.90025244105144	8.52893071141885
H	13.78973837580680	7.22654723775053	7.54690949794007
C	14.09180323073355	7.74281065740147	9.62520218090320
H	13.80550510071556	7.36508099377522	10.61653661044856
H	15.18405639898887	7.70239670370268	9.54342337193769
H	13.77516769585463	8.79302766835739	9.52737878531475
C	11.88895889342353	7.06122448575669	8.57025602294784
H	11.61399551799005	8.10494228838756	8.35641895036401
H	11.37694709548862	6.41872827110522	7.84355561461045
H	11.51923627516222	6.80855000852664	9.56833904098908
C	16.56792036795651	3.14354577208696	11.59525703699637
C	17.03416792927361	3.89953197430487	12.70017111183165
C	17.77554518871081	3.25316803158099	13.69964206391335
H	18.11478740618681	3.82873794502672	14.56169652082439
C	17.66848627190583	1.16550913356642	12.48237627110228
H	17.91712282839532	0.10728431782204	12.39457275608759
C	16.90974295493656	1.77559512029183	11.47409546795884
C	16.76875325317981	5.38435249485375	12.74634232153758
H	17.34789154757096	5.89367510352629	11.96228991848401
H	15.71147426035382	5.61130929551107	12.54329200155282
H	17.05872035119049	5.80356769224315	13.71757018408240
C	16.47801362548113	1.00881454233313	10.24703879568558
H	16.98871330600865	1.38730331595041	9.35032119407270
H	16.69911436119498	-0.06039058598620	10.35221823469446
H	15.40481153805593	1.13190962062904	10.05989827715164
C	18.09079162757815	1.89358041802423	13.59942543809990
C	14.25788970393728	0.14708493898067	12.97836942893249
H	13.28528438283247	-0.33243881458939	13.14202540763562
H	14.28400064137190	0.49620219176495	11.94141979086427
H	15.04892523599772	-0.60603116923587	13.10076303181775
C	9.75790939268411	0.83017270105014	14.07839266640499
H	18.30265606798293	6.34281123746499	6.67791279320684
H	18.66069443887747	1.40384396742707	14.38813425077079
H	9.22289749188491	0.37777396763487	14.91144263997175
H	9.44075152545333	3.01839115510032	6.14413821440006

$(PN)_2Ti{\equiv}NK(18\text{-crown-}6)$

Ti	13.55382014460679	4.20756184066983	11.08491656575912
P	13.43746935033415	2.90842494776068	13.45203631408534
P	13.79757696983499	4.96563543624326	8.51269800084577
N	11.79330930206104	2.93123926494352	10.95958643382622
N	15.69112594321690	3.96356021875630	10.62242501405749
N	13.21241711108372	5.75388186652770	11.63116088261510
C	11.12259058351467	2.34627965818560	12.01287250720310
C	9.81918273005617	1.77551116929293	11.88348280311097
H	9.33356979407257	1.78906829078740	10.91199362928425
C	9.15470802214545	1.21710319845795	12.96951531283806
H	8.15888075112672	0.79870646773375	12.82257881887896
C	11.02365767231998	1.72836882414282	14.39051889311351
H	11.48613282417543	1.71172390882038	15.37508945367450
C	11.72407450550963	2.28465138056823	13.31114332517155
C	13.38779704239141	3.78458952204708	15.11435960247262
H	13.00545145734246	3.04738662324358	15.83236538242288
C	12.40442503369135	4.95976599336525	15.03381710704531
H	12.70452135609214	5.63783965979514	14.22775680426870
H	11.38516954862470	4.61464833459866	14.82942433742668
H	12.40407797774127	5.50670786388241	15.98824909328734
C	14.76918692648815	4.25165988171629	15.59596581612474
H	14.69477058596412	4.61441084679967	16.63196577578455
H	15.52111412891778	3.45584994590617	15.56924697414352
H	15.12936260402730	5.07669113172691	14.97708823099478
C	14.46477867631582	1.35855920994499	13.76500547990429
H	15.49802734801114	1.71229610900846	13.69389120984937
C	14.27429203929344	0.66997051144990	15.12490288423940
H	14.96312116658510	-0.18428762304283	15.19888825922088
H	14.48789304094948	1.33622853150908	15.96841381449574
H	13.25478053373371	0.28291517491535	15.23721974113975
C	11.13549054822714	2.86506992431258	9.69697552410832
C	11.31521695849536	1.73208188156550	8.86615617319788
C	10.67282297744278	1.68882679203379	7.62120977423589
H	10.82296935468044	0.82422148361124	6.97461630895390
C	9.83993883868542	2.73295803246465	7.20816746920258
C	9.62797111797185	3.82469336107217	8.05448871183538
H	8.95730247335133	4.62656292006637	7.74632171143513
C	10.25980340475111	3.90563840307101	9.30361124883222
C	12.14291058644708	0.56474874028774	9.34811231557452
H	12.34840667856127	-0.13656954664145	8.53127625049015
H	13.09312188346009	0.89697118701130	9.77739267399827
H	11.62131470504951	0.02238274274228	10.14956200482874
C	9.97639140482797	5.04323144035162	10.25722203191558
H	9.31995322485044	5.78814271864827	9.79033979738352
H	9.47837547626234	4.66564598959274	11.16128641029953
H	10.90265201978254	5.52585619917031	10.60253507208387
C	16.34587573824378	4.64691035490369	9.62548851235658
C	17.77183449145586	4.75393334539442	9.56223025666172
H	18.36650430705084	4.25741981442804	10.32362264627206
C	18.41088320813596	5.47124296688288	8.55803019074681
H	19.50001750639921	5.52082428155236	8.55390024201648
C	17.67638269115707	6.13938048451089	7.56636312119316
C	16.28171688634816	6.02742761250483	7.58957456464524
H	15.70789997095758	6.52200910448126	6.80879306317402
C	15.60676469675969	5.28784664368257	8.57067725673274
C	13.59807864060601	3.77188536305767	7.06189314641091
H	12.53091067278156	3.51998452987324	7.08582541426854
C	14.40101595386431	2.49816741320567	7.35561183623732
H	15.47761381416212	2.69958992054446	7.30484514619110
H	14.17878845747424	2.10671114625528	8.34959719642457
H	14.15879149512154	1.71735152005551	6.62204847305366
C	13.95139309431063	4.32183113990043	5.67155431205672
H	13.75959493701454	3.54696028215262	4.91514616300253
H	13.35546314893690	5.19947334217186	5.39769005974146
H	15.01329060599392	4.58891092891360	5.60856949164865
C	13.12452300339947	6.57047386454102	7.80174586117999

H	13.70767777932533	6.76184893940785	6.89157936504458
C	13.35090754668825	7.72171451463974	8.78001812837106
H	12.87533964261374	7.48817837198612	9.73950270007415
H	14.41713928351253	7.89716155300521	8.95841124571484
H	12.91449570625055	8.64866493857842	8.38902367136379
C	11.64402686130319	6.48280288489420	7.40246680424632
H	11.35784215836664	7.40129613325447	6.86890625074655
H	11.42656841670843	5.63136292417370	6.75139347988998
H	11.00824202656379	6.39643060598074	8.28689859809011
C	16.51969235782452	3.26646053332906	11.55402109793943
C	16.96268635663985	3.92026725118050	12.72795406012067
C	17.75627282903893	3.22113215609899	13.64784317264592
H	18.08684719069084	3.72636599366486	14.55533444918357
C	17.73316503323347	1.27017003693462	12.22638270737055
H	18.04034660357597	0.24431041023838	12.02438230358094
C	16.93472383020784	1.93948524364445	11.28782607410706
C	16.63697244835804	5.37588999061971	12.93066804506753
H	17.10628096373222	5.99031799834228	12.15516253968895
H	15.55542322411703	5.55693212005941	12.86270903125395
H	17.00457927748019	5.72102528851234	13.90348915617745
C	16.55341272157007	1.27096544992532	9.99074282289285
H	16.99782222135008	1.79504291095823	9.13425517488889
H	16.88867727395929	0.22777651242762	9.97400135525388
H	15.47074620188981	1.29083082473668	9.83271017746366
K	14.41670103348563	8.15048370342729	11.99196524517923
O	11.78958767388693	9.34678757450738	12.76851778194561
O	12.97753213386322	10.32081779476658	10.37874867460967
O	15.70259046563812	10.03388392418914	10.11416927457609
O	17.21852458485466	8.33019597083071	11.66373102107748
O	16.21185110756312	9.07347625342607	14.21233468374343
O	13.56044057242323	8.20017330534736	14.67662879723421
C	11.07307610205264	9.43364970176501	11.52976888297029
H	11.19617437156804	8.51035762868914	10.94183113961589
H	9.99472386839322	9.58667661851650	11.71543073069047
C	11.63290085214827	10.63029599158340	10.78329127412474
H	11.62750879207890	11.50824310927655	11.45003944052782
H	11.01396532170392	10.85392146197252	9.89725033217040
C	13.76901525810985	11.46641365932430	10.05166563102679
H	13.21259334967425	12.15785876619445	9.39453516382854
H	14.04302675589961	12.00917214534985	10.97335680152954
C	15.01334883856631	11.00378570799787	9.31265011897377
H	15.65895041676065	11.87926374402307	9.12265420332416
H	14.74091355200343	10.55308644398224	8.34446764683095
C	17.01187615656741	9.71498389219434	9.61083358434749
H	16.98899891337128	9.56714209952413	8.51853245401835
H	17.69830890250991	10.55156331093969	9.82765249708677
C	17.47649324748864	8.40862750459852	10.25082788666560
H	18.54870327576612	8.26286230537889	10.04208531456062
H	16.93645993411503	7.55979795205620	9.82339664547547
C	17.86927299393071	9.31079555719026	12.48275266171935
H	18.95663698140507	9.31537699383688	12.29129194283810
H	17.46899742759261	10.31720331139672	12.28051105879056
C	17.61748551824964	8.93156955793928	13.93056282324303
H	18.20415011115534	9.58771100734783	14.59728983480011
H	17.93185986569794	7.88976984664647	14.09547813257065
C	15.83123389903495	8.41538965215308	15.43497107870641
H	15.98732508814094	7.32965168672748	15.33568792905453
H	16.44645211908976	8.78152546311650	16.27563048113104
C	14.37467535423246	8.71084066827782	15.73660653562700
H	14.21805388533759	9.79872374350624	15.83994955784875
H	14.11171390693435	8.22869203211707	16.69518393146736
C	12.14889913237934	8.38869737970894	14.90714186165901
H	11.78715475102127	7.65303141879483	15.64155828115051
H	11.97203817884743	9.40344646768992	15.30029511424644
C	11.41195268569716	8.22240526204339	13.59345311038024
H	10.32492988064535	8.23541904327092	13.78490625624960
H	11.68144544443309	7.27637502295629	13.09247260383716
C	18.13623022097610	1.89796158655895	13.40780564277762
C	14.20735226931992	0.37246495942791	12.61443704306566
H	13.19918098967943	-0.05234990967275	12.68549791320021
H	14.29559570263733	0.86034255193783	11.64057900736873
H	14.93837600054837	-0.44629617223643	12.64772368162702
C	9.74093464701688	1.19406349504125	14.24370516127203
H	18.17424028815680	6.70635231186163	6.78225390548020
H	18.74682047144229	1.36220635398257	14.13255015018344
H	9.21809525049602	0.76523828772677	15.09608048715436
H	9.34965530985782	2.68955402529373	6.23692603540173

1

Ti	6.32977921997723	17.05236811562433	10.44446624153174
Ti	6.59559517686764	24.26187775276234	11.16920084437585
K	8.35322025106186	20.57909615089956	10.83167373698117
K	4.56487720403383	20.73541951182706	10.83941493454587
P	5.40193813978109	17.08388636170310	7.89957707253332
P	7.52572503151840	24.75992773853746	8.68308715405657
N	4.29480767067933	16.28643446206268	10.58085411105838
N	8.32006351964107	16.22799216098498	10.11427885096091
N	4.60710873288496	25.13033167829160	11.01948399835174
N	8.62814677316855	24.99012137876808	11.46928928885665
N	6.37882466535798	18.71863845480689	10.63224030607771
N	6.54803347283641	22.59255245392663	11.00871997954833
C	3.31086634915142	16.40625971563618	9.62323953602604
C	1.93982091893407	16.09606143870232	9.88337836330804
H	1.65994947385441	15.75124259975174	10.87519273780302
C	0.96148175691609	16.22750940866505	8.90545130841437
H	-0.07045976887099	15.97946517017865	9.15410668348522
C	1.27969003161814	16.69151878442234	7.62012022170236
C	2.61358102455724	16.99698189433967	7.33821250721686
H	2.86586398388363	17.35976855827240	6.34410840122482
C	3.62835257141641	16.85366362675969	8.29608143150274
C	5.38308601463989	18.61380530189731	6.80169827112994
H	4.61470748120976	18.42133150394840	6.04180465441170
C	4.95744345144089	19.84559402600722	7.60940427382398
H	4.87403939329916	20.71864542463666	6.95084939351964
H	3.98538175968086	19.68213414118443	8.08442083728560
H	5.69986180935951	20.05962776391216	8.38571239227029
C	6.71508184399208	18.85451472319233	6.07723517934618
H	7.51025339631961	19.08930126523126	6.78727561949129
H	7.0356158127433	17.98908729797295	5.49025865442827
H	6.61209883504733	19.70962309946291	5.39407911978991
C	5.77353178275965	15.67553152824373	6.70186258366421
H	6.85738407032937	15.74172764627908	6.55913245633788
C	5.45685084199539	14.34868322148932	7.40905880389145
H	5.86346742468516	13.50706163166544	6.83298212847827
H	5.89542479640439	14.31313965750453	8.40843537755685
H	4.37392495191484	14.20996919730782	7.51195293480164
C	5.09107185208718	15.73099836066839	5.32681994991282
H	4.00309973978004	15.63085188329765	5.41315058784404
H	5.31198859923181	16.65354119743593	4.77918283419311
H	5.44935086084574	14.89228991758440	4.71264069209553
C	3.88398454495086	15.73474486461623	11.83306229450574
C	3.81132524523288	14.33081963655781	12.00297328875534
C	4.08934407668185	13.41111946485175	10.84041346928080
H	5.01700778587162	13.68492448423135	10.32839415749502
H	4.16246785652760	12.37015103124018	11.17411830723489
H	3.29124710480398	13.47928401308931	10.08725928600584
C	3.45136313149405	18.08215313654403	12.66158421861090
H	4.44042147771299	18.45260990445158	12.35806879805287
H	2.75429895993349	18.32947690099072	11.84990956606704
C	9.31603578827842	16.12189109204065	11.06380564121445
C	10.67766914768761	15.85055907419765	10.72420234995781
H	10.94256302843632	15.73091898923632	9.67708026974721
C	11.66436602010582	15.73005801199614	11.69602313318493
H	12.68817969803055	15.51994782080179	11.38590221397303
C	9.01906342583368	16.27577469014325	12.45969813019826
C	7.08716504806701	13.70415773684939	12.78763181011808
H	6.63388128923143	13.89633742670270	11.81264688659838
H	8.16378217554814	13.56201979820171	12.63553506886308
C	8.71018115981446	15.93564514895869	8.77138739768251
C	9.13827146378341	16.97981773549762	7.91847781673211
C	9.48529114392915	16.68890465456734	6.59244932097981
H	9.79723211933734	17.49931634102234	5.93431028275193
C	9.44070329172137	15.37732548780421	6.11239815803491
C	9.07246646426828	14.34063649908538	6.97404051717044
H	9.05186735913239	13.31371611640978	6.60994846682693
C	8.71643063686172	14.59923489383375	8.30513988175058
C	9.26430761115259	18.37713328353879	8.46954871575597
H	9.58816243140242	19.08216034609342	7.69731940071189
H	8.30253572975522	18.70265295894673	8.88833431444191
H	9.99824383237903	18.40653058927280	9.28516234911865
C	8.37965941297818	13.46838183176463	9.24394178094871
H	7.46060144598045	13.67554294812925	9.80041169802947
H	8.25788399277876	12.52713242341561	8.69645239838520
H	9.17074183450801	13.33415709593046	9.99551375807481
C	3.61515370512272	25.04679569288214	11.97512176191845
C	2.25660404281592	25.40186766311141	11.70805402499072
H	1.99153085776582	25.75051811671273	10.71347400923357
H	0.25183651772985	25.60592324693513	12.43438837750847
C	4.21949325390678	25.70624192421729	9.77108424041766
C	4.22319816470856	27.11216749128702	9.60726735633582
C	3.85789471600516	27.65785792324645	8.36909420376027
H	3.88149875172997	28.73957667224321	8.23899024170703
C	3.47268374549481	26.83622257667140	7.30618689109065
C	3.42307085886229	25.45142916376427	7.48759484163001
H	3.09737701555579	24.80683749593901	6.67170368935083
C	3.77816913853662	24.87537261564385	8.71493513920719
C	4.57346122032282	28.00689135914075	10.76939669200235
H	5.50421168895587	27.68985631571462	11.24948393093171
H	4.67929376152648	29.04744847032518	10.44399457851912
H	3.79664388390356	27.96105792125462	11.54542764885235
C	3.63527668297394	23.39339316754149	8.94907558446123
H	3.29915884324026	22.88028203695623	8.04215000270763
H	4.59167161051401	22.96757685880791	9.28057258489678
H	2.89995852832226	23.19937550917647	9.74114329548524
C	9.61536061380481	25.06039641802424	10.50977674075501

C	10.98733309827641	25.30247182321283	10.83016583574835
H	11.26666659726485	25.43584514977511	11.87172778492324
C	11.96784446980782	25.36629360156839	9.84747179659196
H	13.00077752346236	25.55043427250732	10.14305663247040
C	11.65039789967507	25.17742840034899	8.49397449772152
C	10.31561655977291	24.94492200397904	8.15310968285443
H	10.06375193954829	24.79650782843133	7.10522001981558
C	9.29921950879929	24.89525688599666	9.11888752626646
C	7.53913050156930	23.47902168404714	7.30296517989413
H	8.31186550937145	23.81158707988897	6.59837858773211
C	7.94706354195688	22.10940673415791	7.85958183412643
H	7.95808014625403	21.36020749955983	7.05903440152934
H	8.94556522643461	22.14964328247627	8.30624892424008
H	7.22953618857903	21.79676847179107	8.62670506101754
C	6.20765344850412	23.39430436790741	6.54224090993695
H	5.42188898964237	22.97598333285114	7.17460061449685
H	5.86490130972586	24.36866575296158	6.18194886010727
H	6.32481628655772	22.73151707344690	5.67306542418219
C	7.16084500314516	26.38471747302107	7.79830250530741
H	6.07611469472016	26.35882959123193	7.65034158178651
C	7.49367224836969	27.53830232212646	8.75719050525558
H	7.10095709985138	28.48388054621621	8.36020988569878
H	7.05346762173517	27.38041556090115	9.74407657635949
H	8.57813902545456	27.63828741392240	8.88558236601616
C	7.83967396464115	26.60309590752771	6.43764209645849
H	8.92898199524601	26.66942646105542	6.53809911739238
H	7.60428906961226	25.81523800933742	5.71416557908272
H	7.49160352389898	27.55478574700019	6.01098435807527
H	10.23810153193305	22.73761658757770	12.29341430398246
C	8.80917556599922	27.74502750410932	12.32892199277886
H	9.60213055519733	27.84737796110358	11.57439355500877
H	7.88098284491621	27.57065965810447	11.77636751118826
O	11.04296596827760	20.88130584375596	10.44265995155832
C	11.55456626927135	21.91014404609876	9.55981937587547
H	10.76284560292085	22.66165066975812	9.50701032733118
H	12.42335699668925	22.38746053608796	10.03608355610168
C	11.90650756157026	21.40482290060128	8.16267629114209
H	12.24439012273050	22.25213100760105	7.55284072981157
H	12.70853062866865	20.65666769529626	8.18324741914777
H	11.03012616037808	20.96011864481136	7.67613936453154
C	12.05424677298094	19.98479017967857	10.95583284106936
H	12.34949642836920	19.26158023860582	10.17941244758693
H	12.94437066954493	20.57467584360523	11.23077073686275
C	11.48642253065187	19.26348276872970	12.16617340085243
H	12.24313522121376	18.60954199613742	12.61187251131624
H	10.64441522005041	18.61991065715993	11.88791349107983
O	1.88948516066835	20.50321335645528	10.37984985534673
C	0.87654770094177	21.26229089095112	11.07620722918776
H	0.53516071267100	22.10278348523158	10.45116431322328
H	0.01149560068713	20.60562271929656	11.26732914305595
C	1.46768966656884	21.77220767163291	12.37901906902193
H	2.29217142196218	22.47154558273277	12.19906418852678
H	1.82633403856156	20.93721381550489	12.99386427341509
P	7.25624999314736	16.47747060965011	12.91748901800511
P	5.67635158407169	24.30320946375597	13.70733535997272
C	3.44272595906539	13.81040855410617	13.25128803008452
H	3.41258381794203	12.72963903045295	13.38685082137429
C	3.12624107022053	14.65548587706975	14.31816651030203
C	3.14547584538002	16.03965461849950	14.12945911480337
H	2.87437218536467	16.70372717687330	14.94947001934665
C	3.50589846564547	16.59262522059867	12.89280323165405
H	3.12429832223430	18.60717964992948	13.56647528048255
C	11.36358845318149	15.89327844230517	13.05605321539189
C	10.04243707414974	16.17127624590700	13.41387300707813
H	9.80591850092697	16.30752268295779	14.46698429835873
C	7.34133008480559	17.72338581604558	14.32846014663764
H	8.08490528178988	17.32308615325528	15.02923315081023
C	7.84450292941727	19.07617675086953	13.80948646248436
H	7.88205561190404	19.80886163063002	14.62486616148943
H	8.84930888731097	18.98113140678080	13.38558458562079
H	7.16294529898608	19.44812702490350	13.03613925878740
C	6.01370553858328	17.87649623998842	15.08455210248702
H	5.25882366136024	18.35629737639791	14.45793125142361
H	5.60908106350904	16.91801198261861	15.42258125329039
H	6.16733526628079	18.51187260107843	15.96835619711977
C	6.82022045335327	14.85363045308911	13.77152141028756
H	5.73904555372881	14.92918968223903	13.92908577661101
H	6.66426668803879	12.76861234223499	13.17740114410193
C	7.49568744246890	14.57399178322016	15.12241968532601
H	8.58149548950045	14.46789898899215	15.01615237599039
H	7.29266820781776	15.35113469225097	15.86690474085038
H	7.11217855025206	13.62581081901636	15.52622014665665
C	1.27298907549622	25.32018241897740	12.68708683701750
C	1.57295727980777	24.86041747284223	13.97761884358982
C	2.89223785581555	24.50104987208798	14.26277382755125
H	3.12969148145296	24.13836608080039	15.26061877150596
C	3.91329321008839	24.59841187078992	13.30575142227257
C	5.59808123719302	22.78614575197377	14.81955122446474
H	4.86096127377976	23.02689345867341	15.59623914120490
C	5.08509656829196	21.57265127566292	14.03503914590652
H	4.98290254905704	20.70688226195162	14.70085880258242
H	4.10482059214386	21.78258525417501	13.59545914612026
H	5.79104187361861	21.32044687320416	13.23628273239258
C	6.93491726387309	22.48138219388102	15.51017754663946
H	7.69279191757313	22.19083613764818	14.78025707594316
H	7.32254125708934	23.33606133182604	16.07260983129876
H	6.80477078672698	21.64374639058525	16.20995854740172
C	6.11225358250855	25.70203577900717	14.89563211095224
H	7.19345341669098	25.59018936581909	15.03469116732233
C	5.85061627244739	27.03874797937670	14.18524459291460
H	6.28291529818971	27.86536806723248	14.76460672198212
H	6.29789605891738	27.05787851898574	13.18972571150935
H	4.77446997797812	27.21757198648051	14.07353898254694
C	5.43200646462427	25.67996540332733	16.27268240973243
H	4.34814319474672	25.81976988227531	16.18790936681221
H	5.62045992785727	24.75276815661949	16.82460694559914
H	5.82271048772392	26.50818003538960	16.88121048851442
C	9.04372715213252	25.26820064764604	12.80771500236804
C	9.44175570627594	24.20885816993423	13.65637433151870
C	9.82354902175640	24.49059920553799	14.97486966442850
H	10.11449136035613	23.67054899432634	15.63077895444472
C	9.84352084919565	25.80491105190820	15.44949293862359
C	9.50084713805808	26.85357447261065	14.59159382859855
H	9.53175390970502	27.88206743162876	14.95096944323813
C	9.11000704811543	26.60481234817781	13.26866704667400
C	9.50595087999143	22.80536617153649	13.10856970204418
H	9.79244482087243	22.08993496430809	13.88753096889874
H	8.53086371193367	22.52279030241758	12.68870684824985
H	8.72545931621670	28.69132670580878	12.87456339107259
H	11.15808914102166	19.98456107160181	12.92528616682014
H	0.71338499288878	22.32346615638661	12.94948395572614
C	0.99757915575579	20.44312464518136	8.05111052729277
H	0.68550757084252	19.73058946608545	7.27735936565173
H	0.17024717809227	21.14187927941040	8.22581695989364
H	1.85563775599335	21.00991639813648	7.66986517292099
C	1.37545971082787	19.66900662927686	9.31210279755684
H	0.51821522870688	19.09406557778327	9.69131705354534
H	2.17483497960405	18.95507339551674	9.09923228207867
H	10.13388467595660	26.01148820590114	16.47797447994710
H	12.41983438294623	25.21354379780763	7.72615815873209
H	3.20325817322928	27.27298176181795	6.34627698963941
H	0.79997290506267	24.77852539668655	14.73841611925940
H	0.51151317386485	16.80937901513871	6.85938674491586
H	12.13782684718325	15.81414244975472	13.81598257326912
H	9.69811152662130	15.16329238562306	5.07676172605419
H	2.85924546756527	14.23819452670307	15.28737865227440

2

H	9.78498172464794	15.35289765868159	2.13559156716517
H	12.53295779230056	15.32008091032579	7.52805761430483
H	9.56868862951556	14.92565584600510	8.72615608473546
H	12.75447733190973	14.91216406329774	0.92133780641756
C	9.39343751926263	15.48558489123647	6.09125455043856
C	12.93455707873031	15.44006473309106	3.56335045745787
H	9.39386060454121	14.45465445692547	6.46833500533083
H	12.88434185395307	14.41389005030967	3.17670624680943
Ti	11.22888142477103	19.36828044932201	4.84021668005938
Ti	11.27015458256633	24.32496589528329	4.82099318476092
P	9.61093236999346	19.09126908803877	2.75965372300079
P	12.83936089645749	19.04992908652020	6.91743175963850
P	8.67141658654629	24.62894525951211	5.22331674088284
P	13.87233595809783	24.61541316859824	4.41958362437122
N	9.42226564641971	18.37330081666122	5.64468609431385
N	13.00558862571812	18.32341581466464	4.03651895830060
N	10.52193197034110	25.34133206705783	2.99713905917332
N	12.02278819537841	25.35317524859942	6.63514458308368
N	11.26161781946304	22.47714848228234	4.83100821867608
N	11.25112750004832	21.21587786633667	4.83547740890550
C	8.16963967008697	18.49767461573525	5.06796598997416
C	14.26185145833137	18.40519615170303	4.61269775868621
C	6.95802788964513	18.24485273727285	5.77880727140781
C	15.46415751382432	18.10715935119070	3.90337011386555
H	7.01751383595404	17.90154290263884	6.80776811976985
H	15.39340025026611	17.76319678957233	2.87536201553903
C	5.70805959050629	18.47399700929355	5.20560699248279
C	16.72095796152152	18.28848234867672	4.47863254982172
H	4.81100930931186	18.29941800767913	5.80236497962682
H	17.61162111470246	18.07673124977261	3.88427513899598
C	5.59438366625350	18.94228958155020	3.89124927249948
C	16.85038724932899	18.75275431661175	5.79303394532667
C	6.77057635280063	19.13930305459040	3.15410882095405
C	15.68133667497321	18.99550970607687	6.52804396973845
H	6.68885405651803	19.48900561873354	2.12693335077520
H	15.77505344510413	19.34029371850069	7.55592834452988
C	8.03848185965120	18.91596477885491	3.70019229435368

C	14.40671791842185	18.82034758617143	5.97978615486194
C	9.18250953742500	20.35483502214448	1.42459832658603
C	13.31243275056458	20.30358408594510	8.24713222317777
H	8.25776714016911	19.99908230065610	0.95024362912991
H	14.22845931971383	19.92103890521206	8.71767675542925
C	8.89974861243485	21.73286352302607	2.03181875212649
C	13.63534133552042	21.66701186298189	7.62528941698151
H	8.79509323068993	22.48861176004823	1.24341486099145
H	13.77092266118603	22.42822194564024	8.40444702324169
H	7.96131474127375	21.71850693047506	2.58251460646017
H	14.56695446148233	21.61243294323319	7.06524819667517
H	9.68556748250074	22.05668212918307	2.72058975274962
H	12.85454584359851	22.00938961422292	6.93955753482812
C	12.24272387683681	20.40205774079194	9.34488867725625
H	11.31419108014572	20.81626466327208	8.95225334258022
H	12.01354359984878	19.43097900709424	9.79540112719366
H	12.59730819244734	21.07567139605565	10.13897012427734
C	9.59717144175214	17.48257021734418	1.72865006441658
C	12.79359543610276	17.44023094188486	7.94430645017379
H	10.56477825482960	17.49163851246264	1.21641405241678
H	11.82512692550970	17.48205028103355	8.45271407080735
C	9.56536884137875	16.28294337389720	2.68001789744689
C	12.78430039831290	16.24409402402313	6.98732284524354
H	10.30364443220551	16.39095510664180	3.47471386348189
H	12.04848881309292	16.38121393031718	6.19467569469037
H	8.57991276493794	16.18954286906558	3.15190779325709
H	13.76504860729156	16.11887476623095	6.51313968293358
C	8.47814669243719	17.35003149955016	0.68388160021741
C	13.90202746105292	17.26411408873033	8.99353540361993
H	7.48987764071753	17.39303370583713	1.15954321361678
H	14.89340586699990	17.26819915751605	8.52247788460777
H	8.52345945773794	18.12630491613566	-0.08798053569067
H	13.88319463430835	18.04193325221138	9.76509674365293
H	8.56294943116358	16.37568925848921	0.17627578242698
H	13.77626433768757	16.29395597052577	9.50072569406714
C	9.43544799942463	17.86672585418772	6.97940597507872
C	12.97781964765214	17.82947367392538	2.69773631889824
C	9.34768223115779	18.75790199300479	8.08041021876672
C	13.10374162387043	18.72847137530742	1.60706416270579
C	9.40459779169647	18.25996382563988	9.38732718743560
H	9.36124272888427	18.96366498584851	10.21904175882917
C	9.51217163644649	16.88601421729702	9.63438196394464
C	9.52040715551404	16.00188877188965	8.55166583432247
C	12.83889847516477	15.98426330826262	1.10719119629918
C	9.46229331801750	16.46848735850149	7.23123026082806
C	12.90334546757511	16.43559176190095	2.43260026766209
C	9.12631379627369	20.22116739820368	7.82443736154756
C	13.36954538876148	20.18169292282637	1.88082164651353
H	9.06762491264046	20.77938333577195	8.76461984480516
H	13.45344737604699	20.74699883150713	0.94680366432662
H	9.91327548854187	20.65275223310596	7.19102744837391
H	12.58981424803605	20.63115638006465	2.51090265957386
H	8.18828378431390	20.36570898270145	7.28093362352907
H	14.30680197985318	20.29202698227786	2.43448312089319
H	8.48763735876828	15.63975674519781	5.49123081773535
H	13.85146787307298	15.54687022770357	4.15719519378353
H	10.23876950356254	15.60088869516546	5.40507053519581
H	12.10120440377557	15.58753167609507	4.25765218565157
C	9.21215789878816	25.23967809660962	2.55950453802713
C	13.33215003818759	25.25910182296942	7.07464528400017
C	8.82013324453263	25.50793495553667	1.21334802034820
C	13.72467162728154	25.55030124951412	8.41600993566139
H	9.57403977915253	25.84068252149323	0.50549624492407
H	12.97076654728345	25.89383900393585	9.11864602577467
C	7.51190425426615	25.30950524432446	0.77510394256023
C	15.03459380475724	25.36630878019725	8.85521569976189
H	7.26981210012694	25.49719910952540	-0.27249737361156
H	15.27797594255067	25.57533395710874	9.89846347241192
C	6.51976829682053	24.85665093021921	1.65298060284678
C	16.02778091643802	24.90396474149177	7.98338397830181
C	6.86958115488036	24.64147816325239	2.99354062080756
C	15.67672133626330	24.66400450879576	6.64739006280370
H	6.10070728340996	24.30183794442010	3.68478694962627
H	16.44692961733053	24.31890904914917	5.96034515513717
C	8.17256089993025	24.83367204763124	3.46371777806656
C	14.37205584418700	24.84199245534585	6.17615603048263
C	7.41955027608419	23.38574701536705	5.89104053887887
C	15.13420284426788	23.37158249853224	3.76858305313343
H	6.42827115176783	23.77283180422536	5.61810438765312
H	16.12208576831915	23.77114843246769	4.03565844953741
C	7.58499650966853	22.01154354824695	5.23393917848115
C	14.97931858295126	22.00596031327346	4.44764388236381
H	6.95533312649986	21.26627054201265	5.73605886872702
H	15.61105402861316	21.25486995344190	3.95538320718591
H	7.26236262104543	22.04811603259975	4.19474034025063
H	15.30332427115720	22.06316157025151	5.48632855489357
H	8.62061867455233	21.65858778898400	5.25217268587494
H	13.94534403920817	21.64736563269574	4.43694331200860

C	7.47552326666502	23.30505432480460	7.42395559729801
C	15.07923560882390	23.26718124474480	2.23732436601931
H	8.42442316364142	22.88484770096315	7.75631716865427
H	14.13301204775669	22.83617107248831	1.91124392661631
H	7.35621418726564	24.28231810544319	7.90239375428502
H	15.19301324971697	24.23850914284454	1.74515301478276
H	6.67417272781667	22.64354784821422	7.78483193600876
H	15.88482739556675	22.60543455445930	1.88630202856969
C	7.98862426651037	26.24044020591174	5.98879734411292
C	14.54263187000787	26.22550247327528	3.64032840343242
H	8.35483074807214	26.21164818649345	7.01962838851847
H	14.18021439847365	26.18423533925829	2.60871575440421
C	8.63266569792576	27.43497679609153	5.28029421276471
C	13.88395250883146	27.41970166998200	4.33648162120652
H	8.45948324597504	28.36100506108665	5.84744269023914
H	14.05198518457604	28.34269947837555	3.76307075131543
H	9.70961895953468	27.29715676400095	5.17980709110177
H	12.80755235490258	27.27326760277889	4.43185241030566
H	8.21845194183594	27.55678048557674	4.27219958752860
H	14.29104719949461	27.55276425105905	5.34596270957449
C	6.46055452772357	26.40014498589156	6.00932703149835
C	16.06892925767728	26.39916268873444	3.62329120041073
H	6.04622081199940	26.37000758610092	4.99325827272093
H	16.47997367321044	26.38283302051579	4.64099367444171
H	5.96382387301962	25.62911549453332	6.60852090798576
H	16.57420337593744	25.62631371476429	3.03340675261088
H	6.19972676260487	27.37663756275705	6.44821270701572
H	16.32240835983294	27.37346503545099	3.17542031900899
C	11.45002648044787	25.84628801082665	2.03636962146112
C	11.09161003212757	25.85536508753438	7.59403289518144
C	12.13510969652078	24.95627475639252	1.16853293531253
C	10.42336990213982	24.96323041705391	8.47248623363519
C	9.48548435897188	25.45667943777677	9.38698412802073
H	8.96542789574303	24.75206020175584	10.03653781783333
C	9.21387715550490	26.82713818380703	9.47713090098807
C	12.59771347741408	27.71106948534153	0.95693244661757
C	9.92610218448502	27.71366998998115	8.66422763381294
H	12.75774400717494	28.78700493187677	0.86856171882717
H	9.75162802775322	28.78797893766282	8.74441543985172
C	11.65015674232158	27.24461386184790	1.87833403640547
C	10.87466337535267	27.25211260177957	7.74132252149484
C	11.79502005553591	23.49395283442347	1.19659882055435
C	10.78227353596673	23.50511999626056	8.45169633081784
H	12.43213652361869	22.92784321006496	0.50939064599790
H	10.15970321569903	22.93600694984870	9.14956137418470
H	11.88178288060288	23.07298965833609	2.20730261870876
H	10.69050800462711	23.07551867067347	7.44488264721404
H	10.75333681720208	23.34731615961829	0.89618044759882
H	11.82912309243806	23.37387640675965	8.74259142514242
C	10.82693027338595	28.22967054400454	2.66726481069754
C	11.68284999031506	28.24014446223320	6.94060181881101
H	11.08518938625664	29.25952188921010	2.38847861681298
H	11.40380485421546	29.26918975527799	7.20160563764069
H	9.75440304989491	28.07635636271865	2.49378738174065
H	12.75734758428034	28.10989093106516	7.12147295839494
H	10.98298967206504	28.11770529195249	3.74527008676048
H	11.53486744693439	28.10756453867347	5.86396088179043
C	10.26191281798932	20.41352596652191	0.33322483323742
H	9.93239750016810	21.09131285151344	-0.46798320543842
H	11.19919078029572	20.80379777441637	0.73006849616675
H	10.46680380208916	19.43340599844474	-0.10881756105405
C	13.07212397993601	25.45467159321521	0.25546842543509
H	13.60480260640183	24.75191170225606	-0.38584685620313
C	13.32633308202122	26.82771725473993	0.15539775802278
C	13.04132562963053	18.24524785657037	0.29498567142790
C	12.88770161780396	16.87830451108313	0.03361513661133
H	13.11649830985913	18.95475576459473	-0.52946482906420
H	9.57324158371352	16.51068721864783	10.65531601441629
H	4.62095510458329	19.14503609151289	3.44665661474691
H	12.82271734216907	16.51521365412230	-0.99151670871892
H	17.83023719411346	18.91643615206654	6.23962132178707
H	8.46931021480485	27.19851647277581	10.18044702181617
H	17.04842349604527	24.74037970504257	8.32663589026049
H	14.06974801742409	27.20339827838427	-0.54687436960193
H	5.50121086648938	24.67962233210948	1.31019161277030

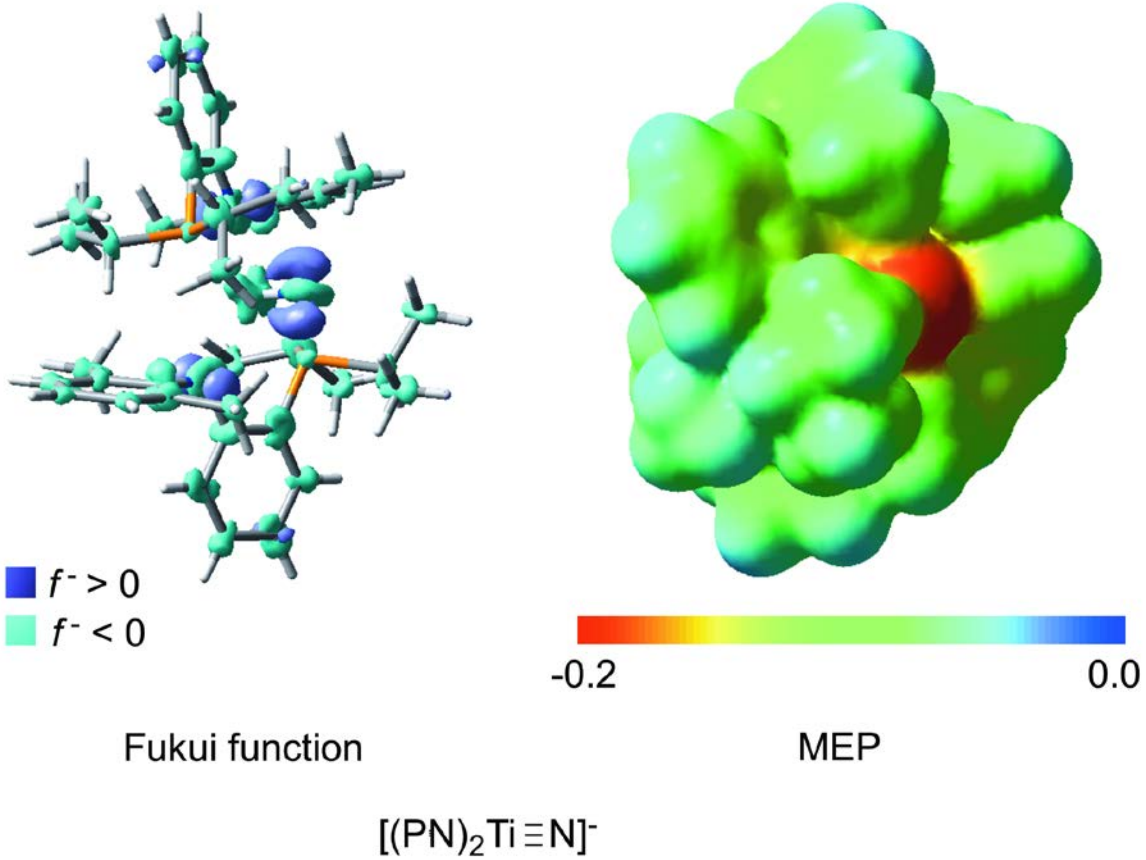

Figure S19. Nucleophilic Fukui function, f^-, and molecular electrostatic potential, MEP, for $[(PN)_2Ti{\equiv}N]^-$ characterizing soft (purple regions) and hard (red sites) electrophilic reactivity of the titanium-nitride functionality, respectively,.

Crystallography Details. Suitable single crystals for X-ray analysis of **2-8** were placed on the end of a Cryoloop coated in NVH oil. The X-ray intensity data collection was carried out on a Bruker APEXII CCD area detector using graphite-monochromated Mo-K[illegible] radiation (λ = 0.71073 Å) at 100(1) K. Preliminary indexing was performed from a series of thirty-six 0.5° rotation frames with exposures of 10 seconds. Rotation frames were integrated using SAINT,[12] producing a listing of non-averaged F^2 and $\sigma(F^2)$ values which were then passed to the SHELXTL[13] program package for further processing and structure solution. The intensity data were corrected for Lorentz and polarization effects and for absorption using SADABS.[14] All calculations were performed using SHELXS[15] and SHELXL.[16] The structures were solved by Patterson and Fourier transform methods. All reflections were used during refinement. Non-hydrogen atoms were refined anisotropically and hydrogen atoms were refined using riding models with exception for **7**. For **4**, two crystallographically independent, but chemically equivalent molecules are present in the asymmetric unit. For **5**, ^{i}Pr groups of NP^iPr_2 were disordered over two on the crystallographycally special position. The thermal ellipsoids were fixed by SHELXL restraint commands,

DELU and SIMU. Disordered pentane molecule was located with 0.5 occupancies and a negative PART number, and refined with a rigid group model. For **6**, one site occupied by hexane was identified in the asymmetric unit. This site was considerably disordered and was treated by SQUEEZE as a diffuse contribution.[17, 18] In the resulting void space, a contribution of 134 e^- per unit cell was found and taken to represent one hexane in the asymmetric unit, giving one pentane molecule for each Ti complex. For **7**, one site occupied by THF was identified in the asymmetric unit. This site was also treated by SQUEEZE as a diffuse contribution, resulting in a contribution of 186 e^- per unit cell to give one THF molecule for each Ti complex. This data was treated as a two-component crystal data. One component with 0.8 occupancies was refined as a terminally bound parent imide complex **7**. The hydrogen atom of the imide ligand was located from the difference map and refined isotropically. The second component was considerably an insertion product in which the imide ligand has inserted into the arm of the PN ligand. The phosphorus atom and corresponding ^{i}Pr groups were refined with 0.2 occupancies, but the hydrogen atom corresponding to the parent imide hydrogen in **7** could not be located from the difference map due to its low occupancy. These results were checked using the IUCR's CheckCIF routine. The alerts in the output are related to the disordered groups and crystal solvents.

Solid state ^{15}N NMR Details. Solid-state ^{15}N NMR spectra were recorded under the cross polarization (CP) magic-angle spinning (MAS) condition on a Bruker Avance-600 NMR spectrometer (14.1 T) operating at the ^{1}H and ^{15}N Larmor frequencies of 600.17 and 60.81 MHz, respectively. The Hartmann-Hahn matching condition was established with a solid $^{15}NH_4NO_3$ sample. High-power ^{1}H decoupling (70 kHz) was applied during data acquisition. A 4-mm Bruker MAS probe was used with sample spinning frequencies between 5.0 and 14.5 kHz. A relaxation delay of 2 s and a contact time of 2 ms for CP were used. Powder samples were packed into a ZrO_2 rotor (4-mm o.d.) in a glove box. All ^{15}N chemical shifts were referenced to that of NH_3(liq) (δ = 0 ppm) by using solid $^{15}NH_4NO_3$ as a secondary ^{15}N chemical shift reference (δ = 23.8 ppm). Spectral simulations were performed using DMFit.[19]

References

1 F. Neese, ORCA—An ab initio, Density Functional and Semiempirical Program Package Version 3.0.1 (2013)
2 A. D. Becke, *Phys. Rev. A*, 1988, **38**, 3098.
3 C. T. Lee, W. T. Yang and R. G. Parr, *Phys. Rev. B,* 1988, **37**, 785.
4 D. A. Pantazis, X. Y. Chen, C. R. Landis and F. Neese, *J. Chem. Theory Comput.*, 2008, **4,** 908.
5 a) K. Eichkorn, F. Weigend, O. Treutler and R. Ahlrichs, *Theor. Chem. Acc.*, 1997, **97**, 119. b) K. Eichkorn, O. Treutler, H. Öhm, M. Häser and R. Ahlrichs, *Chem. Phys. Lett.*, 1995, **240**, 283.
6 K. Eichkorn, O. Treutler, H. Öhm, M. Häser and R. Ahlrichs, *Chem. Phys. Lett.*, 1995, **242**, 652.
7 a) E. van Lenthe, E. J Baerends and J. G. Snijders, *J. Chem. Phys.*, 1993, **99**, 5648.; b) C. van Wullen, *J. Chem. Phys.*, 1998, **109**, 392.
8 E. J. Baerends, J. A., D. Bashford, A. Børces, F. M. Bickelhaupt, C. Bo, P. M. Boerrigter, L. Cavallo, D. P. Chong, L. Deng, R. M. Dickson, D. E. Ellis, M. van Faassen, M. Fan, T. H. Fischer, C. Fonseca Guerra, A. Ghysels, A. Giammona, S. J. A. van Gisbergen, A. W. Gotz, J. A. Groeneveld, O. V. Gritsenko, M. Grüning, F. E. Harris, P. Harris, P. van den Hoek, C. R. Jacob, H. Jacobsen, L. Jensen, G. van Kessel, F. Kootstra, M. V. Krykunov, E. van Lenthe, D. A. McCormack, A. Michalak, M. Mitoraj, J. Neugebauer, V. P. Nicu, L. Noodleman, V. P. O. Osinga, S. Patchkovskii, P. H. T. Philipsen, D. Post, C. C. Pye, W. Ravenek, J. I. Rodriguez, P. Ros, P. R. T. Schipper, G. Schreckenbach, M. Seth, J. G. Snijders, M. Solà, M. Swart, D. Swerhone, G. te Velde, P. Vernooijs, L. Versluis, L. Visscher, O. Visser, F. Wang, T. A. Wesolowski, E. M. van Wezenbeek, G. Wiesenekker, S. K. Wolff, T. K. Woo, A. L. Yakovlev and T. Ziegler, V. U. *ADF2013.01*, Amsterdam (The Netherlands).
9 S. H. Vosko, L. Wilk, Nusair, M. *Can. J. Phys.* 1980, **58**, 1200.
10 J. P. Perdew, J. A. Chevary, S. H. Vosko, K. A. Jackson, M. R. Pederson, D. J. Singh and C. Fiolhais, *Phys. Rev. B*, 1992, **46**, 6671.
11 M. Krykunov, T. Ziegler, E. van Lenthe, *J. Phys. Chem. A*, 2009, **113**, 11495 and references therein.
12 *SAINT*; Bruker AXS Inc.: Madison, WI, USA. 2009.
13 *SHELXTL*; Bruker AXS Inc.: Madison, WI, USA, 2009.
14 G. M. Sheldrick, *SADABS*; University of Gottingen: Germany, 2007.
15 G. M. Sheldrick, *Acta Crystallogr.* 2008, **A64**, 112.
16 $R_1 = \Sigma||F_o| - |F_c|| / \Sigma |F_o|$, $wR_2 = [\Sigma w(F_o^2 - F_c^2)^2/\Sigma w(F_o^2)^2]^{1/2}$, $GOF = [\Sigma w(F_o^2 - F_c^2)^2/(n - p)]^{1/2}$;where n = the number of reflections and p = the number of

parameters refined.

17 A. L. Spek, *Acta Crystallogr.* 2009, **D65**, 148.
18 P. van der Sluis and A. L. Spek, *Acta Crystallogr.* 1990, **A46**, 194.
19 D. Massiot, F. Fayon, M. Capron, I. King, S. Le Calvé, B. Alonso, J.-O. Durand, B. Bujoli, Z. Gan, G. Hoatson, Modelling one- and two-dimensional solid-state NMR spectra. *Magn. Reson. Chem.*, 2002**, 40**, 70-76.
